# Serial monitoring of circulating tumor DNA in patients with primary breast cancer for detection of occult metastatic disease

**DOI:** 10.15252/emmm.201404913

**Published:** 2015-05-18

**Authors:** Eleonor Olsson, Christof Winter, Anthony George, Yilun Chen, Jillian Howlin, Man-Hung Eric Tang, Malin Dahlgren, Ralph Schulz, Dorthe Grabau, Danielle van Westen, Mårten Fernö, Christian Ingvar, Carsten Rose, Pär-Ola Bendahl, Lisa Rydén, Åke Borg, Sofia K Gruvberger-Saal, Helena Jernström, Lao H Saal

**Affiliations:** 1 Division of Oncology and Pathology, Department of Clinical Sciences, Lund UniversityLund, Sweden; 2 Lund University Cancer CenterLund, Sweden; 3 SCIBLU Genomics, Department of Clinical Sciences, Lund UniversityLund, Sweden; 4 Department of Pathology, Skåne University HospitalLund, Sweden; 5 Department of Radiology, Skåne University HospitalLund, Sweden; 6 Department of Surgery, Lund University and Skåne University HospitalLund, Sweden; 7 Department of Immunotechnology, Lund UniversityLund, Sweden; 8 CREATE Health Strategic Centre for Translational Cancer Research, Lund UniversityLund, Sweden

**Keywords:** breast carcinoma, circulating tumor DNA, early detection, liquid biopsy, metastasis

## Abstract

Metastatic breast cancer is usually diagnosed after becoming symptomatic, at which point it is rarely curable. Cell-free circulating tumor DNA (ctDNA) contains tumor-specific chromosomal rearrangements that may be interrogated in blood plasma. We evaluated serial monitoring of ctDNA for earlier detection of metastasis in a retrospective study of 20 patients diagnosed with primary breast cancer and long follow-up. Using an approach combining low-coverage whole-genome sequencing of primary tumors and quantification of tumor-specific rearrangements in plasma by droplet digital PCR, we identify for the first time that ctDNA monitoring is highly accurate for postsurgical discrimination between patients with (93%) and without (100%) eventual clinically detected recurrence. ctDNA-based detection preceded clinical detection of metastasis in 86% of patients with an average lead time of 11 months (range 0–37 months), whereas patients with long-term disease-free survival had undetectable ctDNA postoperatively. ctDNA quantity was predictive of poor survival. These findings establish the rationale for larger validation studies in early breast cancer to evaluate ctDNA as a monitoring tool for early metastasis detection, therapy modification, and to aid in avoidance of overtreatment.

See also: **TM af Hällström *et al*** (August 2015)

## Introduction

Breast cancer is the most common malignancy and leading cause of cancer-related death in women worldwide; once the tumor has metastasized, it is essentially an incurable disease (Jemal *et al*, 2011). The difficulty in curing metastatic breast cancer may be in part because metastatic spread is usually detected only after the deposit has grown large enough to be palpable, cause overt clinical symptoms, or be identified by imaging. In patients with primary (non-metastatic) breast cancer at diagnosis, the risk of subsequent metastatic relapse is greatest within 2 years after primary surgery (Cheng *et al*, 2012). However, an estimated 50% of recurrences are diagnosed > 5 years after surgery (Early Breast Cancer Trialists’ Collaborative Group, 2005), indicating that occult metastatic dissemination can have a protracted subclinical period. Earlier detection of metastatic breast cancer may be clinically beneficial. A reasonable assumption is that identification of recurrent disease at the earliest moment will allow for initiation of auxiliary therapies against a nominal tumor burden that has accumulated fewer oncogenic events. So far, this assumption has been tested without success, most likely because modalities and biomarkers that lack sufficient sensitivity and/or specificity have been utilized thus far (Lippman & Osborne, 2013). For example, whereas circulating tumor cells (CTCs) may carry additional prognostic information in primary breast cancer (Lucci *et al*, 2012; Rack *et al*, 2014), available evidence does not support the use of imaging, serum protein markers, and CTCs for routine monitoring after primary surgery (Khatcheressian *et al*, 2013; Theriault *et al*, 2013). At the same time, many breast cancer patients are likely being overtreated; that is, they may in fact be cured by locoregional treatment and unnecessarily enduring the side effects of systemic therapies. For these reasons, improved surveillance methods to determine occult tumor burden (or lack thereof) in the primary breast cancer setting are still highly desirable (Lippman & Osborne, 2013).

Clinical monitoring of minimal residual disease is routinely performed in several hematological malignancies with known pathognomonic chromosomal rearrangements, for example by serial quantification of TEL-AML1 or BCR-ABL fusion-gene chromosomal translocations in acute lymphoblastic leukemia and chronic myelogenous leukemia, respectively (Dolken, 2001). In cancer patients, tumor-derived DNA (termed cell-free circulating tumor DNA; ctDNA) can be found in the blood circulation and usually comprises a small fraction of the total circulating DNA (Jung *et al*, 2010). Circulating DNA is rapidly degraded into short fragments, and the quantity of ctDNA appears to be related to tumor progression (Stroun *et al*, 1989; Diehl *et al*, 2008; Yung *et al*, 2009; Jung *et al*, 2010; Leary *et al*, 2010; McBride *et al*, 2010; Diaz *et al*, 2012; Dawson *et al*, 2013; Murtaza *et al*, 2013; Bettegowda *et al*, 2014; Newman *et al*, 2014). Therefore, ctDNA “liquid biopsy” analysis is an attractive biomarker for noninvasive monitoring of tumor growth, response, and spread (McDermott *et al*, 2011). Until recently, assays for ctDNA have been infeasible for most solid cancers due to a paucity of recurrent mutations for interrogation as well as the practical and economical hurdles of enumerating tumor-specific aberrations on a per-patient basis.

Advances in deep-sequencing technology now enable comprehensive cataloguing of tumor-specific (somatic) chromosomal rearrangements and mutations at an ever-decreasing cost (Meyerson *et al*, 2010). Recent studies have shown that breast cancer genomes may harbor from a few to several hundred rearrangements and mutations per tumor (Shah *et al*, 2009; Stephens *et al*, 2009, 2012; Banerji *et al*, 2012; Cancer Genome Atlas Network, 2012; Ellis *et al*, 2012; Nik-Zainal *et al*, 2012). In contrast to somatic point mutations, in which the identical mutation can be present across many tumors, tumor types, and individuals (for example *PIK3CA* hot-spot mutations), chromosomal rearrangements are inherently highly tumor specific and can serve as unique genetic “fingerprints” of an individual tumor (Leary *et al*, 2012). Serial measurement of ctDNA using various methods has shown encouraging results for several solid cancer types (Diehl *et al*, 2008; Yung *et al*, 2009; Leary *et al*, 2010; McBride *et al*, 2010; Diaz *et al*, 2012; Misale *et al*, 2012; Newman *et al*, 2014), and in the metastatic breast cancer setting, measurement of ctDNA dynamics compares favorably to the serum protein marker CA 15-3 and CTCs (Dawson *et al*, 2013).

**Figure 1:**
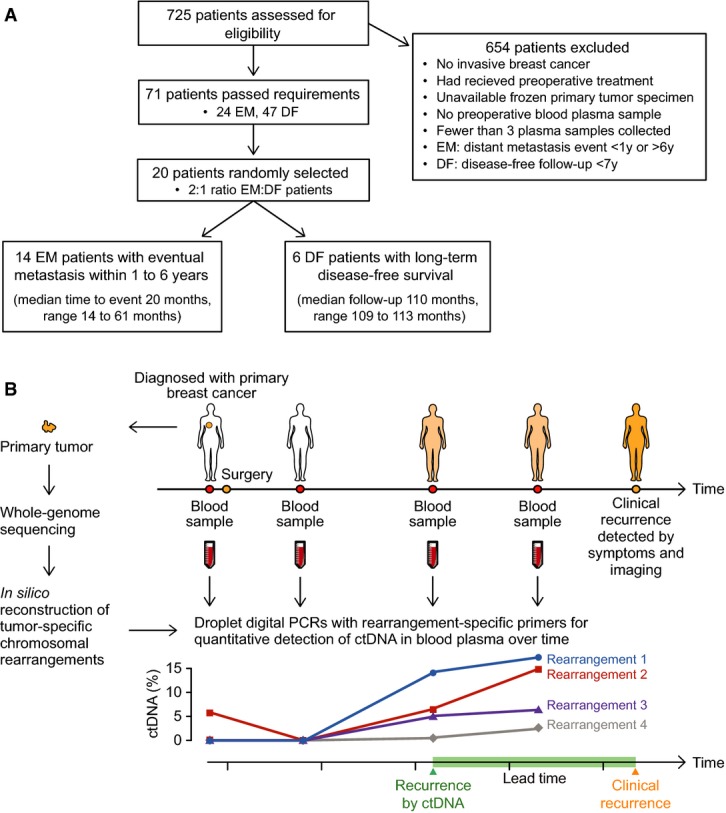
Analysis of personalized ctDNA biomarkers in primary breast cancer Patient flow diagram indicating patient selection criteria. EM = eventual metastasis; DF = long-term disease-free.

Here, we tested in patients with primary breast cancer and long-term follow-up the hypothesis that monitoring of tumor-specific chromosomal rearrangements in cell-free circulating DNA can detect occult metastatic disease following primary surgery and serve as a sensitive, specific, and thus potentially clinically useful noninvasive biomarker in the adjuvant setting (Fig1).

Study schema. For 20 women with primary breast cancer, patient- and tumor-specific chromosomal rearrangements were determined through whole-genome sequencing of 21 tumor tissue specimens (one patient had bilateral tumors). Genomic fusion sequences were bioinformatically reconstructed, and selected rearrangements were validated as somatic. Personalized droplet digital PCR assays were used to quantify rearranged DNA sequences in the cell-free circulating DNA isolated from 93 patient blood plasma samples taken serially during the clinical course. ctDNA results were then compared to clinical endpoints.

## Results

### Enumeration of tumor-specific chromosomal rearrangements

**Figure 2:**
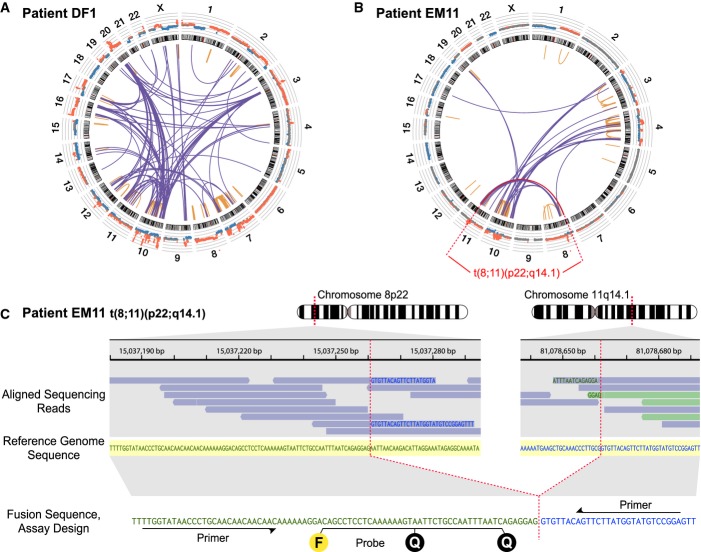
Identification of chromosomal rearrangements and personalized assay design Low-coverage whole-genome sequencing of the primary tumor was used to enumerate chromosomal rearrangements. Shown are results for patient DF1, with inter- and intra-chromosomal rearrangements plotted as a Circos diagram (Krzywinski *et al*, 2009). Chromosomes 1–22 and X are ordered in the outer circle. From the outside, concentrically, are plotted the DNA copy number estimations from the whole-genome sequencing data and the chromosome ideograms. The orange intra-chromosomal and blue inter-chromosomal arcs in the center indicate chromosomal rearrangements supported by two or more paired-end reads.

Twenty patients enrolled in the Breast Cancer and Blood Study (BC Blood, Sweden) (Borgquist *et al*, 2013), an ongoing prospective study at Lund University since 2002, were included in the present investigation for retrospective analysis of ctDNA (Fig1A). Six patients had long-term disease-free survival (9.2 years median follow-up; termed DF patients), and 14 had eventual diagnosis of clinical metastasis from 1.2 to 5.1 years after primary surgery (termed eventual metastatic [EM] patients) (Table1). For each patient, a sample of the primary tumor, a normal tissue sample, and 3–6 blood plasma samples that were collected during the clinical course were available. First, to identify tumor-associated chromosomal rearrangements that could serve as biomarkers, whole-genome sequencing (WGS) was performed on DNA isolated from 21 primary breast tumors (patient EM6 had bilateral primary breast cancers). On average, 93 million DNA fragments were sequenced per tumor (range 54–160 million), yielding a mean genome sequence coverage of 5.3-fold (range 1.8–12.9) and mean physical coverage of 15.6 (range 9.2–28.2) (Supplementary Table S1). We developed an analysis pipeline incorporating our SplitSeq computational method to identify inter- and intra-chromosomal rearrangements using an approach that scanned for paired sequence reads where the two reads aligned to discordant positions in the human genome, or individual reads in a read pair that contained juxtaposed sequences from two disparate genomic regions. Chromosomal rearrangements supported by two or more sequenced fragments could be detected in all primary tumors, and on average, 92 rearrangements were identified per tumor (range 21–305) (Fig2, Supplementary Fig S1 and Supplementary Tables S1 and S2). There was no significant difference in sequence coverage or frequencies of chromosomal rearrangements detected between EM patients and DF patients (Mann–Whitney test), and the numbers of detected rearrangements for these 21 cases are similar to other studies of primary breast tumors (Stephens *et al*, 2009; Banerji *et al*, 2012; Nik-Zainal *et al*, 2012).

**Table 1:** Patient and tumor characteristics

Patient ID	Age at primary diagnosis (years)	Tumor size (mm)	Lymph node status (positive/total)	Distant metastasis at diagnosis	ER status	PR status	HER2 status	Nottingham Histological Grade	Time to recurrence (months)	Time to last follow-up or death^b^ (months)
EM1	42	33	0/2	No	Positive	Positive	Negative	3	20.0	29.4^b^
EM2	57	28	1/17	No	Positive	Positive	Negative	2	40.0	55.2^b^
EM3	78	20	6/17	No	Positive	Positive	Negative	3	16.1	17.9^b^
EM4	34	28	0/2	No	Positive	Positive	Negative	2	31.8	99.0
EM5	61	12	0/5	No	Positive	Positive	Negative	1	48.8	97.1^b^
EM6	62	Right: 28	2/13	No	Positive	Positive	Negative	2	61.3	87.3
		Left: 55	1/12		Positive	Positive	Negative	3		
EM7	55	22	0/2	No	Positive	Negative	Amplified	2	18.9	59.3^b^
EM8	67	22	2/12	No	Positive	Negative	Negative	2	13.9	33.2^b^
EM9	50	18	0/1	No	Positive	Positive	Negative	3	36.0	54.7^b^
EM10	64	45	1/14	No	Positive	Negative	Negative^a^	2	17.7	33.2^b^
EM11	59	20	16/18	No	Positive	Positive	Negative^a^	3	13.9	32.5^b^
EM12	53	37	4/10	No	Positive	Positive	Negative	2	16.2	33.5^b^
EM13	69	25	0/4	No	Positive	Negative	Negative^a^	2	43.9	58.7^b^
EM14	47	19	1/18	No	Negative	Positive	Amplified	2	20.0	46.7^b^
DF1	58	15	0/2	No	Positive	Positive	Negative	3		108.7
DF2	37	20	0/3	No	Positive	Positive	Negative	3		111.7
DF3	56	19	0/1	No	Positive	Positive	Negative^a^	2		109.9
DF4	46	13	0/1	No	Negative	Negative	Negative^a^	2		110.4
DF5	54	15	0/2	No	Positive	Positive	Negative^a^	3		109.5
DF6	58	18	0/2	No	Positive	Negative	Negative^a^	2		113.2

All patients analyzed are women.

a Clinical HER2 analysis not performed. HER2 status determined from gene copy number derived from whole-genome sequencing results.

b Time from primary diagnosis to death.

Circos diagram for patient EM11. Plots for all patient tumors are shown in Supplementary Fig S1.

One example rearrangement from patient EM11, indicated in red in (B), with identification of the exact fusion sequence between chromosomes 8p22 and 11q14.1. Aligned sequencing reads are highlighted in blue when its read pair aligns concordantly on the same chromosome or in light green if its read pair aligns on another chromosome. Within each sequencing read, nucleotide bases with exact match to the reference sequence (shown in the middle with yellow shading) are not printed. Mismatching bases are shown in blue if matching to 11q14.1 and green if matching to 8p22. At the bottom, the personalized dual-labeled probe and primers designed for this validated rearrangement are illustrated. F denotes the fluorescent molecule and Q the two quenching molecules.

### Selection and validation of rearrangements

To account for possible intra-tumoral heterogeneity, and since it is not possible to know *a priori* which rearrangements in the primary tumor will be part of derivative metastatic clone(s), candidate rearrangements were selected such that a range of apparent copy number states (in other words, a range of number of supporting reads) were represented for each patient tumor. Our strategy was to design assays for ∼10 rearrangements per primary tumor and select additional rearrangements in the event of assay failure or validation as not somatic. In summary, for each of the 237 selected candidate rearrangements, one assay was designed and tested by conventional PCR across the breakpoint junction in tumor and normal DNA from the same patient. Of 197 informative assays (83%; 7–17 per tumor), 167 (85%) were confirmed to be somatic by PCR (Supplementary Tables S3 and S4). Of these, due to limitations on the available plasma volumes and our desire to perform replicate analyses, four to six rearrangements per tumor were selected (again to reflect a variety of copy number states) and the corresponding probe was synthesized for droplet digital PCR (ddPCR) analysis of patient plasma samples. Probe assay success rate was high, with 113 of 122 (93%) validating for ddPCR (Supplementary Table S4).

### Optimization of droplet digital PCR

**Figure 3:**
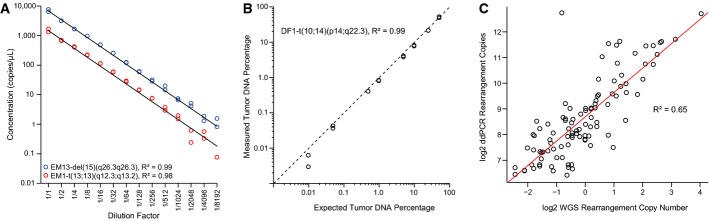
Performance of droplet digital PCR (ddPCR) method Dilution series for two tumor-specific rearrangements, patient EM13-del(15)(q26.3q26.3) and patient EM1-t(13;13)(q12.3;q13.2), starting with input of 20 ng of the respective patient’s primary tumor DNA in each ddPCR, and diluting twofold in the series as indicated (*x*-axis). Experiments were performed in duplicate. Linear regression lines are plotted in black, and goodness of fit statistics (*R*^2^) were calculated.

In ddPCR, the PCR with input DNA and target sequence-specific fluorescent probe and primers is partitioned into thousands of nanoliter-sized reaction droplets. Following thermocycling, successful amplification of the target cleaves the fluorescent molecule from the specific probe, thereby unquenching the fluorophore (Fig2C). Each droplet is read as either containing amplifiable target sequence (positive fluorescence above a threshold) or not, yielding a binary (digital) readout. Because the distribution of zero, one, two, or more amplifiable targets into droplets is a random process, the fraction of positive droplets to total droplets can be Poisson-corrected to derive a highly quantitative estimate of the number of amplifiable molecules that were present in the input sample (Hindson *et al*, 2011). We optimized a ddPCR method for measurement of circulating DNA that employs a universal touchdown PCR thermocycling protocol for increased specificity. For quantification of tumor-specific rearrangements, we determined our ddPCR method to be highly linear over at least 3 orders of magnitude and able to discriminate somatic mutant rearranged sequences down to 0.01% tumor DNA content (one rearranged sequence per 10,000 wild-type sequences) (Fig3A and B). Importantly, zero tumor-specific rearrangements were detected by our method in over 2.7 million negative control DNA droplets analysed, corresponding to > 200 control ddPCR reactions that in total interrogated more than 2.5 million normal haploid genome equivalents (i.e. zero rate of false-positive signals).

Observed percentages by ddPCR of a tumor-specific chromosomal rearrangement, patient DF1-t(10;14)(p14;q22.3), in admixtures of tumor and normal DNA of varying amounts from 50% down to 0.01% tumor DNA content (total DNA input fixed at 200 ng). Concentrations of the tumor-specific rearrangement and the control region in chromosome 2p14 were used in the calculations for amounts of tumor and total DNA, respectively. The black diagonal dashed line indicates the ideal correlation line (*y *= *x*). The *R*^2^ was calculated for the linear regression line (not plotted). All axes are on log scales.

Correlation between whole-genome sequencing (WGS) rearrangement copy number estimates and the number of copies in 40 ng primary tumor DNA as measured by ddPCR. Axes on log_2_ scales. The *R*^2^ was calculated for the linear regression line (drawn in red).

### Quantification of ctDNA in serial plasma samples

Circulating cell-free DNA was isolated from 93 plasma samples for the 20 patients. The number of fragments of each tumor-specific chromosomal rearrangement was quantified in the circulating DNA by ddPCR. Each tumor-specific rearrangement assay was run in duplicate and included positive (primary tumor DNA) and negative (matched normal DNA) controls, and on average, 25,704 (SD 2,320) droplets were analyzed per assay per plasma sample. As expected, the relative copy numbers of rearrangements were well correlated between the WGS analysis and ddPCR analysis of primary tumor DNA (*R*^2^ = 0.65; Fig3C). A ddPCR assay targeting a non-rearranged normal region of chromosome 2p14, which rarely undergoes copy number alteration in breast cancer (Jonsson *et al*, 2010), was used to estimate total circulating DNA (both tumor and normal cell derived). The average number of amplifiable 2p14 control region fragments was 1,908 copies/ml plasma (range 280–8,960) (Supplementary Fig S2 and Supplementary Table S5). There was no significant difference in the number of 2p14 control region fragments per ml plasma between EM and DF patients within the preoperative time-points nor when comparing across all time-points (Mann–Whitney *U*-test). Tumor-specific rearrangements were detected in 29 plasma samples corresponding to 13 EM patients, and the fractional quantity was calculated as the measured rearrangement divided by the measured 2p14 control region. In these 29 samples, ctDNA levels (taking the maximal value if more than one rearrangement was detected in a sample) ranged from 1.4 to 72.4% (mean 19.3%), and the concentration of rearranged fragments ranged from 38 to 2,617 fragments/ml plasma (mean 552 fragments/ml plasma) (Supplementary Table S5). The lowest ctDNA level detected in our patient material was 0.45%.

**Figure 4:**
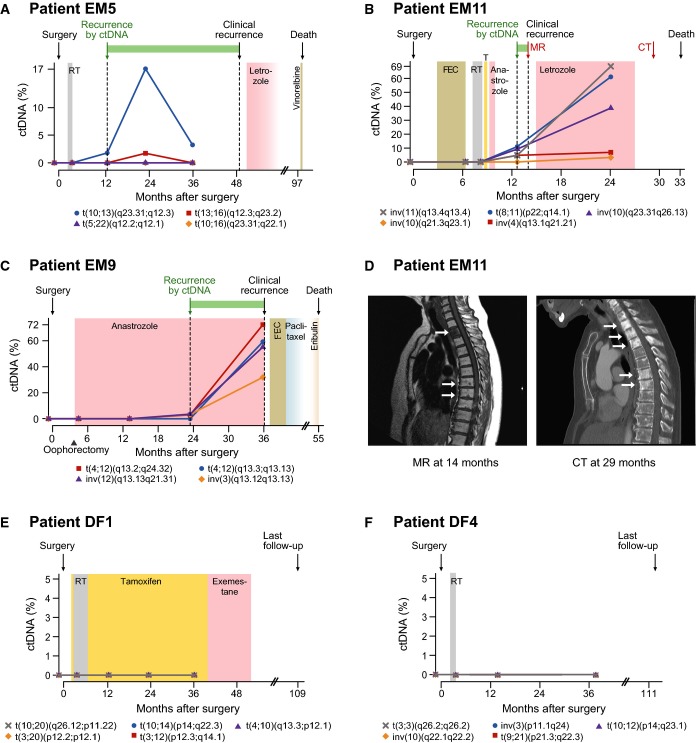
Monitoring multiple tumor-specific chromosomal rearrangements in circulating DNA A–C Plasma levels of circulating tumor DNA (ctDNA), quantified using ddPCR, for three patients with known eventual recurrence. Specific rearrangements are indicated by colored markers and labeled according to cytogenetic nomenclature (t denotes translocation, inv is inversion, and del is deletion). The recurrence by ctDNA time-point is defined as the earliest follow-up plasma sample (after surgery) with ctDNA detected at a level greater than 0% (compared to total cell-free circulating DNA) for at least one rearrangement. All relevant clinical events are indicated above by arrows, time gain by ctDNA-based detection is indicated by a green horizontal bar, and radiation (RT), endocrine, and cytotoxic treatments are indicated by colored shading. T = tamoxifen; FEC = fluorouracil, epirubicin, and cyclophosphamide. See Supplementary Fig S2 for ctDNA time-course plots with clinical annotations for all patients.

Among the 14 EM patients with known eventual clinical recurrence, 13 patients had positive ctDNA levels for one or more follow-up plasma time-points and only patient EM3 had undetectable ctDNA (Fig4A–C and Supplementary Fig S2). Conversely, none of the patients with long-term disease-free survival had detectable ctDNA at any time-point after surgery (Fig4E and F and Supplementary Fig S2). Thus, our noninvasive blood test for metastasis during follow-up had a sensitivity of 93% and specificity of 100% (95% confidence intervals [CI] 66–100% and 61–100%, respectively) for discrimination of EM versus DF status. Of note, ctDNA was detected in the presurgical plasma sample for four of 20 patients (20%; EM2, EM8, EM12, EM14); all four of these patients had eventual recurrent disease.

D Correlative magnetic resonance (MR; T1 weighted) and computed tomography (CT) imaging for patient EM11 corresponding to the red arrows in (B). In the MR, low T1 signal (dark) is present in the entire second thoracic vertebra and as punctate lesions in several vertebrae in the middle thoracic spine. The CT 15 months later shows sclerosis (white) in multiple additional thoracic vertebrae, consistent with progression of metastatic disease.

E, F ctDNA plots for two patients with long-term disease-free survival.

**Figure 5:**
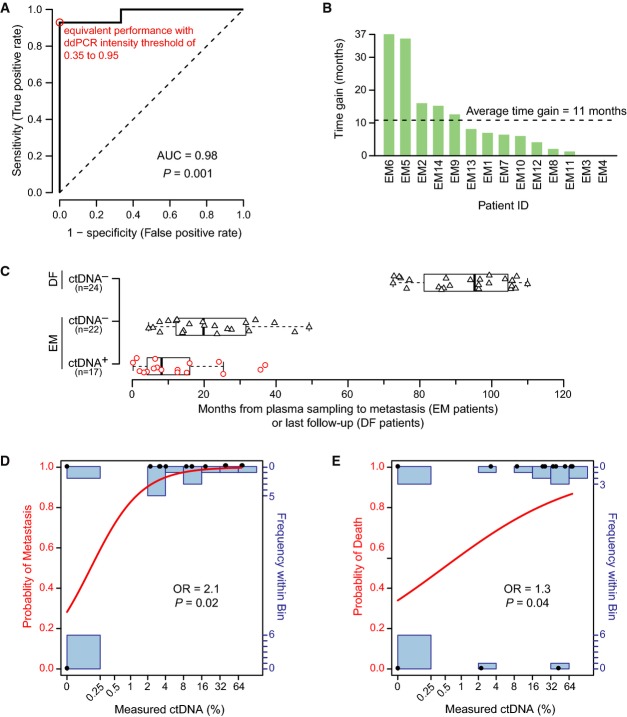
ROC analysis, time gain, and clinical outcome Receiver operating characteristic (ROC) curve analysis. The area under the curve (AUC) as a measure of the postsurgery classification accuracy to discriminate between 6 long-term disease-free (DF) and 14 eventual metastasis (EM) patients based on ctDNA is 0.98 (95% CI 0.75–1.00; *P *=* *0.001, two-sided Mann–Whitney *U*-test). The sensitivity and specificity were maximal (red circle) at all ddPCR relative fluorescence intensity thresholds between 0.35 and 0.95 (on a normalized scale from 0 to 1). The dashed line indicates a hypothetical test with performance no better than random.

For each ddPCR assay, a uniform threshold of 0.5, i.e. at 50% of the normalized range of intensity values between the positive and negative control droplets, was used. Because the discriminatory accuracy of our ctDNA test could be influenced by the fluorescent intensity threshold used in dichotomizing a ddPCR droplet as positive or negative, we performed receiver operating characteristic (ROC) curve analysis wherein the intensity threshold was varied incrementally (see Supplementary Methods; Supplementary Fig S3). This analysis indicated our test to have a high accuracy for postoperative discrimination of EM versus DF patients with an area under the curve of 0.98 (95% CI 0.75–1.00; *P *=* *0.001, Mann–Whitney *U*-test) and an equivalent performance across a wide range of fluorescence intensity thresholds, from 0.35 to 0.95 (Fig5A). Thus, the ddPCR signals were robust and distinct.

Time gained by ctDNA-based detection of recurrence in advance of clinically detected recurrence for all patients with clinical recurrence. For 12 out of 14 EM patients, ctDNA-based recurrence preceded clinical recurrence (time gain greater than zero).

Boxplots indicating the time from a positive (red circles) or negative ctDNA plasma sample (black triangles) until an event, metastasis or last follow-up, for EM and DF patients. Box indicates the interquartile range (IQR), thick bar indicates the median, and whiskers extend to values within 1.5 times the IQR.

Fitted curve from logistic regression with metastasis as endpoint. Measured ctDNA percentage and actual outcomes are indicated by black dots, the modeled probability is given by the red curve (left axis), and the number of measured data points in each bin is indicated by the blue bar graphs (right axis). Logistic regression odds ratio (OR) of 2.1 (95% CI 1.3 to infinity; *P *=* *0.02, Wald test) is for each doubling of ctDNA.

Fitted curve from logistic regression with death as endpoint. OR of 1.3 (95% CI 1.03-1.9; *P *=* *0.04, Wald test) is for each doubling of ctDNA.

### ctDNA and clinical course

Circulating tumor DNA showed dynamic changes across serial plasma samples for 13 of 20 patients (65%) (Supplementary Fig S2 and Supplementary Table S5). Three representative examples of changes in ctDNA levels during the clinical course are highlighted below. Due to the favorable clinicopathological features of patient EM5 (12 mm primary invasive ductal carcinoma, wide margins, no positive lymph nodes, histological grade 1, estrogen and progesterone receptor positivity, and HER2 negativity), she received postoperative radiotherapy and no systemic adjuvant therapy. Clinical metastasis was detected at 49 months after primary surgery; however, our ctDNA-based method detected molecular recurrence at 13 months, providing a potential earlier diagnosis of metastatic cancer by 3 years (Fig4A; Supplementary Table S6). Analysis of ctDNA identified one tumor-specific rearrangement between chromosomes 10q and 13q in the plasma sample from 12-month follow-up; this and a 13q-16q rearrangement were detected at 24 months, and 10q-13q at 3 years. Two additional rearrangements (5q-22q and 10q-16q) were not detected at any time-point, indicating that these may not have been present in the cancer clone(s) that seeded the metastasis or that they were present below our level of detection.

Patient EM11 displayed complex circulating tumor DNA dynamics. She was diagnosed with stage III invasive ductal carcinoma, hormone receptor-positive and high-grade histopathology, and received radiotherapy as well as several adjuvant systemic therapies due to intolerance (Fig4B). Molecular recurrence was detected at 13 months via positive detection of four out of five rearrangements in her circulating DNA. Clinical recurrence was diagnosed at 14-month follow-up due to bone pain and confirmed by magnetic resonance imaging (Fig4D), and she received letrozole therapy. At the 24-month follow-up time-point, however, three of five rearrangements increased in abundance by fourfold to 14-fold, one chromosome 4q inversion remained stably low, and a fifth rearrangement (inversion on 10q) could be detected. This is consistent with partial response of the tumor clone containing the 4q inversion but inherent or acquired resistance to letrozole by one or more subclones containing the other four rearrangements. Computed tomography (CT) of the spine at 14 months showed progressive disease, consistent with ctDNA quantification (Fig4D). Similarly, for patient EM9, three out of four tumor-specific chromosomal aberrations indicated molecular recurrence at 23-month follow-up, preceding clinical detection by 13 months, and during ongoing anastrozole therapy (Fig4C). All four rearrangements increased dramatically at the 36-month follow-up time-point, coincident with confirmed distant metastases in the brain and liver by CT.

Interestingly, our sequencing analysis of the bilateral tumors of patient EM6 confirmed that they were two independent primaries with no clonal relatedness (Supplementary Table S2). Furthermore, ctDNA analyses indicated that the right-side tumor gave rise to the occult metastatic disease that was detectable by ddPCR at 2-year follow-up (37 months prior to clinical recurrence; Supplementary Table S6), whereas there was no ctDNA evidence of metastatic disease arising from the left primary tumor (Supplementary Fig S2).

### ctDNA as a predictive factor

In patients with known eventual clinical metastasis, ctDNA-based molecular detection of occult metastasis preceded the clinical diagnosis in 12 of 14 patients (86%), with an average lead time window of 11 months (range 0–37 months) (Fig5B; Supplementary Table S4). Furthermore, a positive ctDNA blood test was always eventually followed by clinical detection of metastasis with a median time from a positive ctDNA test to clinical metastasis of 8 months (Fig5C). Among EM patients, a negative ctDNA test occurred for 13 of 14 patients at least once (patient EM14 has only positive time-points), with median time from a negative ctDNA blood test to a clinical metastasis of 20 months (Fig5C). However, for all patients but one, the negative ctDNA tests were followed by a positive test (patient EM3 had undetectable ctDNA at all time-points). For the two EM patients (EM3, EM4) with exclusively negative ctDNA results prior to clinical metastasis (Supplementary Fig S2), the time interval from the preceding negative ctDNA test to clinical metastasis was 4.5 and 12 months, respectively (Fig5C), indicating that narrower time intervals of ctDNA testing could be considered in future prospective studies.

Finally, we found ctDNA level to be quantitatively predictive of poor clinical outcome. Whereas none of the conventional univariable biomarkers (tumor size T, node status N, histological grade, ER, PR, HER2, or Nottingham Prognostic Index) were associated with outcome using logistic regression in this limited patient series, ctDNA level was a significant predictor of poor disease-free survival (odds ratio (OR) of 2.1 for each doubling of ctDNA level, 95% CI 1.3 to infinity; *P *=* *0.02; Fig5E) as well as poor overall survival (OR 1.3 for each ctDNA doubling, 95% CI 1.03–1.9; *P *=* *0.04; Fig5F) (Supplementary Table S7).

## Discussion

We studied cell-free circulating DNA in patients with primary breast cancer and show that ctDNA monitoring is accurate for the detection of occult metastasis. Metastasis could be detected by ctDNA in plasma for 13 of 14 patients and in none of the 6 patients with long-term disease-free survival. Moreover, ctDNA-based detection preceded clinical detection of metastasis for 86% patients with an average lead time of 11 months, and ctDNA was found to be a significant predictor for poor disease-free and overall survival. As far as we are aware, this study is the first to demonstrate that ctDNA monitoring can herald clinical detection of metastasis by months to several years and that ctDNA level, even when measured in the setting of primary breast cancer, is associated with significantly increased risk of poor outcome. Our results are in line with a recent report analyzing ctDNA in metastatic breast cancer patients using similar methods (Dawson *et al*, 2013) and are a significant finding given that ctDNA levels are considerably lower in earlier stage disease and thus inherently more difficult to detect than after clinical diagnosis of metastatic disease (Bettegowda *et al*, 2014). Together, these data provide support for the evaluation of ctDNA in the adjuvant setting in larger prospective studies to address several important questions. For example, it should be ascertained in clinical trials whether tailoring secondary adjuvant therapy by ctDNA monitoring can increase the rate of long-term breast cancer cure. Second, although other modalities have not shown a clinical benefit of early detection of occult metastasis, our results suggest that ctDNA may have the performance characteristics needed for earliest and accurate detection. This prompts for evaluation of whether and to what extent detection of occult metastasis by a ctDNA monitoring can improve outcomes. Furthermore, as part of a “watchful waiting” approach, additional inexpensive yet sensitive and specific molecular surveillance by liquid biopsies could help enable a reduction in the “overtreatment” of patients with low-risk breast cancer.

Our method combines low-pass whole-genome sequencing with quantitative ddPCR-based personalized rearrangement analysis of plasma ctDNA and can be performed across dozens of liquid biopsies per patient for < €1,000 in reagents and < €50 per time-point, currently making it more much cost effective than approaches where sequencing of each liquid biopsy time-point is performed. Our analysis can also be achieved within a clinically useful time frame. In practice, candidate rearrangements could be identified and personalized ddPCR assays validated within 1 month of tumor biopsy, and a panel of ddPCR tests on patient plasma samples can be performed within 1 day. Multiple chromosomal rearrangements, supported by variable numbers of sequencing reads (including those nearby copy number aberrations which may be under positive selection), were chosen for plasma analysis to overcome the potential issue of intra-tumoral heterogeneity, where only a subclone comprising a varying fraction of the primary tumor gives rise to the metastatic growth(s). The chance for a false-negative result, where metastatic disease is present but never detected by ctDNA analysis, will decrease with each additional genomic aberration tested. In a WGS analysis of matched primary and metastatic breast cancers from the same patients, typically over 50% of chromosomal rearrangements present in the primary tumor can be found in its distant metastatic tumor, indicating that most genomic rearrangements occur relatively early during tumorigenesis and can be stable fingerprints for an individual’s breast cancer (Tang and Gruvberger-Saal, manuscript in preparation). Determining the optimal criterion for candidate rearrangement selection and how many to monitor per patient/tumor are matters deserving additional study. Here, we chose to monitor four to six selected rearrangements per tumor due to limited volumes of plasma, which nevertheless was sufficient to detect metastatic disease in 13 out of 14 patients. Patient EM3 (Supplementary Fig S2), the only EM patient where we did not detect any ctDNA, had the fewest number of plasma samples (three compared to a median number of five samples per patient); therefore, we believe that increasing volume and frequency of plasma samples would be more beneficial than increasing the number of rearrangements tested per case. Our patient results for time to an event following a positive or negative ctDNA plasma sample suggests an interval of ∼4–6 months between sampling may be reasonable, at least during the first few years of follow-up.

Our ddPCR-based method has similar analytical performance characteristics to other recently described methods for the analysis of circulating DNA, such as nested real-time PCR (McBride *et al*, 2010), digital PCR (Dawson *et al*, 2013), personalized analysis of rearranged ends (Leary *et al*, 2010), targeted deep sequencing of mutated genes of interest (Dawson *et al*, 2013), or direct deep sequencing of circulating DNA (Leary *et al*, 2012). We show our ddPCR method to be highly reproducible, linear, and able to detect 1 mutant target within 10,000 wild-type sequences. Importantly, our method capitalizes on the unique juxtaposition of sequences formed by chromosomal rearrangements and thus is less prone to false-positive signals compared to methods that use a preamplification step of the circulating DNA and/or assays that must discriminate between single-base differences amid wild-type and mutated alleles (Beaver *et al*, 2014). Our method’s zero false-positive rate for the detection of somatic rearrangements in over 2.5 million control normal haploid genomes compares exceedingly well to other methods and is a critical feature needed for clinically useful monitoring of patients with primary cancer where ctDNA fractions are low.

Although our results demonstrate the promise and benefits of ctDNA monitoring in primary breast cancer, there are several limitations. One, this proof-of-principle study was limited to 20 patients. Larger validation studies will be important to further clarify the utility of ctDNA monitoring in early-stage breast cancer and within the molecular subtypes. The availability and quantity of archival frozen plasma as well as the specific time-points of their collection also limited us. In the current configuration, in which cell-free DNA was isolated from 0.5 ml plasma and 4% of this was input per replicated ddPCR reactions (for four to six rearrangements per case), we estimate our method to be sensitive to detect one amplifiable target DNA molecule in 40 μl of plasma (approximately 25 targets per ml of plasma). The sensitivity of our method to detect exceedingly low counts of target ctDNA could be improved linearly by increasing the amount of input DNA into ddPCR reactions, by multiplexing, by preamplification, and/or by isolating circulating DNA from a larger volume of plasma. For example, greater amounts of analytical material from 5 to 50 ml plasma would allow for an improved limit of detection of our method by at least one order of magnitude and to 1 target ctDNA molecule per 5 ml plasma or better. In the prospective setting, there would be the opportunity to better control the blood plasma collection procedures and time-points and take larger volume samples. Therefore, the sensitivity and apparent lead time advantage for occult metastasis detection reported herein may in fact be an underestimation.

Recently, Bettegowda and colleagues reported that ctDNA was detected in a single time-point for 10 of 19 patients with localized breast cancer when inputting cell-free DNA isolated from 2 to 5 ml plasma; but no association with outcome was possible (Bettegowda *et al*, 2014). In our study of patients with primary breast cancer, ctDNA could be detected in the presurgical plasma sample for 4/20 patients and all four had eventual recurrent disease. Although the sample size is small, and given the limitation of available plasma discussed above, the variation between patients in presurgical levels of ctDNA is intriguing and suggests that presurgical levels could serve as a potential prognostic factor deserving further study. In theory, ctDNA should be present in all patients prior to primary surgery. The limited plasma availability, and desire to analyze four to six rearrangements per time-point, likely impacted our preoperative detection rate. Indeed, oversampling for 17 patients with remaining presurgery cell-free DNA was possible using a single assay tested in at least 3 additional ddPCR reactions, which increased the presurgery detection rate to 9/20 (45%). Circulating tumor DNA monitoring might be feasible for the measurement of minimal residual disease at a time-point shortly after primary surgery; prospective studies with optimized plasma collection schedule and much larger plasma volumes will be required to evaluate this important question.

We have shown that ctDNA monitoring can herald clinical metastasis by months to years and that ctDNA is a quantitative predictive factor for poor outcome in the primary breast cancer setting. The future of breast cancer medicine is personalized therapies and precision care. For this to become a reality, noninvasive and accurate methods for monitoring of breast cancer progression and response to treatment will be necessary within the neoadjuvant, adjuvant, and metastatic settings. Patient monitoring using noninvasive assays for ctDNA is proving to be a realistic means to discern biologically and clinically relevant information and shows great promise for incorporation into routine clinical management.

## Materials and Methods

### Ethics statement

The study was approved by the Regional Ethics Committee at Lund University including permission to publish de-identified clinical images (DNR 75-02, 37-08, 658-09, 58-12, 379-12, and 227-13). Trained health professionals provided written and oral information and all patients signed written informed consent in accordance with the WMA Declaration of Helsinki and the U.S. Department of Health and Human Services Belmont Report.

### Patients

Patients enrolled in the Breast Cancer and Blood Study (BC Blood, Sweden) (Borgquist *et al*, 2013), an ongoing prospective study at Lund University since 2002, were included in the present investigation for retrospective analysis of ctDNA. As shown in Fig1A, patients were identified based on the following criteria: non-metastatic (stage I–III) breast cancer at initial diagnosis who received no neoadjuvant therapy, availability of frozen primary tumor specimen, frozen presurgery and two or more follow-up plasma samples collected during clinical course, and either clinically detected distant metastasis 1–6 years after diagnosis (termed eventual metastatic [EM] patients) or long-term disease-free survival > 7 years at last follow-up (termed DF patients). Out of 725 patients assessed, 24 EM and 63 DF patients passed eligibility requirements. From these, 20 patients were randomly selected 2:1 with respect to EM:DF categories. This sample size with multiple time-points per patient was considered to be sufficient to demonstrate the feasibility of ctDNA monitoring and test the hypothesis that occult metastasis can be detected by ctDNA analysis. Fourteen EM patients (first metastasis detected clinically at 14–61 months following diagnosis, median 20 months) and 6 DF patients (disease free at last follow-up, 109–113 months after diagnosis, median 110 months) were studied (Table1 and Fig1). The 20 patients were diagnosed between November 2002 and May 2007, received the standard of care, and were followed according to Swedish National Guidelines as well as additional structured follow-up as part of the BC Blood Study: patients met with a research nurse for study questionnaires (aimed at assessing symptoms and change in medication) and serial blood collection at specified time-points: prior to primary surgery and at approximately 3- to 8-, 12-, 24-, and 36-month follow-up time after primary surgery, and for biennial questionnaires thereafter. This was in addition to the routine clinical follow-up, which for patients not receiving chemotherapy consisted of clinical visits and mammography at follow-up years 1, 2, and 3 after primary surgery, and then by mammographic surveillance in the national screening program; and for patients receiving chemotherapy consisted of a clinical evaluation after completing chemotherapy and followed by yearly clinical visits up through year 5, and then by mammographic surveillance. If any of the follow-up modalities indicated symptoms or signs of metastatic disease, appropriate imaging and confirmatory workup was performed per standard clinical practice. All cancer therapies are indicated for each patient in Supplementary Fig S2. For all patients included herein, all collected blood sample time-points were analyzed, and study results were blinded to the clinic. In all parts (sequencing, circulating DNA isolation, and ddPCR), patients were analyzed in random order without regard to clinical parameters and the ddPCR data were analyzed in an automatic fashion blinded to outcome and operator (detailed below).

### Whole-genome sequencing analysis

Primary tumor specimens were snap-frozen immediately after surgery and stored at −80°C in the South Swedish Breast Cancer Group tumor bank. The tumor DNA isolation method is described in the Supplementary Methods. Whole-genome paired-end Illumina sequencing libraries were constructed from tumor DNA sheared to a median insert size of 500 bp, sequenced on our laboratory HiSeq 2000 instruments, and aligned to the human reference genome GRCh37 (Supplementary Table S1). Matched normal genomic DNA was isolated for all patients from whole blood. For three of the included patients as well as seven unrelated patients, normal genomic DNA samples were also sequenced and used to filter germline and false-positive rearrangements arising from errors in the human reference genome sequence and from regions of unreliable mappability. Chromosomal rearrangements were identified (Supplementary Fig S1 and Supplementary Table S2) and the exact rearrangement fusion sequence reconstructed using our bioinformatics pipeline SplitSeq (Supplementary Methods). PCR validation is described below and in the Supplementary Methods.

### Plasma DNA isolation and ddPCR

Blood samples were collected from patients in EDTA tubes and were centrifuged to separate plasma from peripheral blood cells within 2 h of collection, and the fractions were frozen at −80°C. Total cell-free circulating DNA was isolated from 0.5 ml plasma using the QIAamp UltraSens Virus DNA kit (Qiagen) with protocol modifications. For selected rearrangements, polymerase chain reaction (PCR) primers and a double-quenched fluorescent 5′–3′-exonuclease hydrolysis probe were designed (mean amplicon size, 101 bp; range 63–155 bp) (Supplementary Table S3). For a subset of the rearrangements confirmed somatic using touchdown PCR with rearrangement-specific primers and primary tumor DNA or matched normal DNA as input (Supplementary Methods; Supplementary Table S4), the probe was synthesized (Integrated DNA Technologies) and the quantitative assay validated using a Bio-Rad QX100 droplet digital PCR (ddPCR) instrument using primary tumor DNA and matched normal DNA as controls. A ddPCR assay (Supplementary Table S3) targeting a 132-bp non-rearranged normal region of chromosome 2p14, which rarely undergoes copy number alteration in breast cancer (Jonsson *et al*, 2010), was used to estimate total circulating DNA (both tumor- and normal cell derived). For ddPCR, four to six tumor-specific rearrangement assays were analyzed, wherein 4% (4 μl) of the isolated cell-free DNA (corresponding to 20 μl plasma) was input in each assay reaction and the absolute count of the target sequence was measured (Hindson *et al*, 2011). Primary tumor DNA and matched normal DNA were used as positive and negative controls, respectively, for every personalized rearrangement assay in every ddPCR run, and a no-template control (water) was used as a negative control for the 2p14 control assay. All rearrangement reactions were run in duplicate. Detailed methods are presented in the Supplementary Methods.

### ddPCR data normalization

To enable an unbiased, uniform, and outcome- and operator-blinded automatic evaluation of ddPCR data, droplet fluorescent intensity measurements of each assay were normalized to a relative scale ranging from 0 to 1 by scaling to the negative control and positive control droplet intensities, for each assay, using custom scripts (see Supplementary Methods and Supplementary Fig S3). Droplets with a relative intensity ≥ 0.5 were defined positive (receiver operating curve characteristic analyses were performed to assess discriminatory accuracy at all thresholds; see below). The number of fragments per μl input purified circulating DNA (*C*_*Vi*_) was calculated from the number of positive droplets *P*, total number of droplets analyzed *T*, droplet volume *V*_*d*_ (0.91 × 10^−3^ μl), ddPCR volume *V*_*r*_ (including PCR mix, primers, probe, input DNA), and volume of purified circulating DNA input into the reaction *V*_*i*_, using the formula 
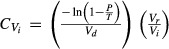
. A plasma sample was defined to be positive for ctDNA if one or more of the target tumor-specific rearrangements in the sample had a molecular count greater than zero by ddPCR analysis. To control for possible variability in the efficiency of plasma DNA isolation or degradation of cell-free circulating DNA during long-term storage of plasma, for each rearrangement, ctDNA level was estimated as a percentage of total circulating DNA by dividing the quantity of measured rearrangement by the quantity of the 2p14 control region.

### Receiver operating characteristic (ROC) curve analysis

Because the fluorescent intensity threshold used in calling ddPCR droplets positive or negative may influence the accuracy of ctDNA-based monitoring for occult disease, we applied a ROC curve analysis. In this analysis, the droplet intensity threshold, for every assay, was incrementally varied from 0 to 1 in 0.1 steps and applied to the normalized data for all samples, defining negative droplets (below threshold) and positive droplets (above threshold). At each threshold, the concentration of each rearrangement was calculated across all time-points and the rearrangement with the highest concentration was used to represent each time-point as this was thought to be most clinically relevant. Thus, ctDNA was represented and analyzed using a single covariate. Based on this, a patient was classified either as recurrence positive if one or more plasma samples during the follow-up period were positive for ctDNA, or as recurrence negative if all plasma samples during the follow-up period were negative for ctDNA. The predicted recurrence state was then compared with the known true recurrence state obtained from the clinical records in order to determine true-positive (TP), true-negative (TN), false-positive (FP), and false-negative predictions (FN). Sensitivity was calculated as TP/(TP+FN), and specificity was calculated as TN/(TN+FP). Sensitivity was plotted against 1–specificity for each threshold, producing a ROC curve. The area under the curve was calculated using the R package *ROCR* (Sing *et al*, 2005).

### Statistical analyses

All statistical calculations were done in R v2.14.1. Confidence intervals for sensitivity, specificity, and area under the ROC curve were calculated based on the Clopper–Pearson exact binomial distribution method using the R package *binom* v1.1-1 (see Supplementary Methods). Except for the logistic regression odds ratios (see below), the Mann–Whitney test for significance was utilized throughout because the data types are not normally distributed and this test makes no assumption on the distribution. All *P*-values and confidence intervals calculated are two-sided except for the confidence interval for specificity (one-sided 95% confidence interval since the proportions are estimated to 1).

### Logistic regression

To determine the influence of ctDNA level and primary diagnosis clinical parameters (Table1) on the risk of clinical metastasis and of death, we carried out univariable logistic regression analyses. Postsurgical plasma ctDNA percentage levels were used as a continuous covariate by taking, for each patient, the most recent plasma sample time-point prior to an outcome event, and for each time-point, using the rearrangement with the maximal ctDNA percentage value as this was thought to be most clinically relevant. Due to quasi-complete separation of ctDNA level between DF patients (Fig5D, lower black dots) and EM patients (Fig5D, upper black dots), we employed Firth’s penalized likelihood approach (Firth, 1993) that allows reliable estimation also for separated data (Heinze, 2006). Since we assumed that, for example, a 10-unit increase in ctDNA percentage from 0 to 10% may have a different prognostic implication than an increase of the same magnitude from 50 to 60%, we allowed for nonlinear effects of ctDNA levels on the risk. Log_2_-transformation minimized the summed Akaike information criteria (see Supplementary Methods); therefore, log_2_-transformed ctDNA percentage was used as covariate, and accordingly, the resulting odds ratios are for each twofold increase in percentage ctDNA (e.g., from 1 to 2%, or 3 to 6%). The primary diagnosis clinical parameters of tumor size (T3, > 5 cm, versus T1, ≤ 2 cm, and T2, 2–5 cm), number of positive lymph nodes (N1, 1–3 positive, N2, 4–9 positive, and N3 > 9 positive nodes versus N0, none), Nottingham histological grade (G3 versus G1 and G2), estrogen receptor status (ER negative versus ER positive), progesterone receptor status (PR negative versus PR positive), and HER2 status (HER2 positive versus HER2 negative) were each used as single covariates in univariable logistic regression analyses with respect to the outcome variables, clinical recurrence, and vital status at last follow-up. No other candidate variables were considered. For patient EM6 with bilateral breast cancer, the variables for the left-side tumor with worse clinical prognostic features were used (Table1). Analyses were carried out using the R package *brglm* (Kosmidis, 2013), with the statistical significance of estimated odds ratios evaluated by the Wald test.

### Data deposition

The raw unprocessed droplet digital PCR data and normalized data have been deposited in the Dryad Digital Repository (http://datadryad.org) with identifier doi: 10.5061/dryad.b6928 (http://dx.doi.org/10.5061/dryad.b6928). Due to patient privacy, the whole-genome sequencing data, which may contain personally identifiable genetic variation and disease-associated alleles, are not publicly available.

## For more information

Author’s Web site: http://tinyurl.com/saalgroup.

The paper explained Problem Breast cancer is the most common cancer affecting women and a leading cause for cancer-related death. Despite our best medical treatment, late metastatic recurrences are common. Unfortunately, metastatic breast cancer is usually diagnosed only after it has become symptomatic, and by this time, it is essentially incurable. On the other hand, a significant number of patients with non-metastatic breast cancer may be “overtreated” and subject to unnecessary side effects of systemic therapies when they are in fact cancer free. It may be possible, with a highly sensitive and specific molecular method to quantify circulating tumor DNA, to detect asymptomatic metastatic recurrences early or to determine a cancer-free state, noninvasively, using a blood test. Results We have used whole-genome sequencing of breast cancers to identify tumor-specific chromosomal rearrangements that serve as molecular “fingerprints” of each patient’s cancer. This is followed by droplet digital PCR-based quantification of tumor-specific rearranged DNA molecules in patient blood samples collected at various time-points during their clinical follow-up. Here, we identify for the first time that ctDNA monitoring provides a sensitive method for early detection of asymptomatic metastatic recurrence in patients diagnosed with primary breast cancer and that the presence and quantity of ctDNA is predictive of poor outcome in this key patient group. Patients with long-term disease-free survival had no detectable ctDNA at any time-point after surgery. Impact Our study shows that ctDNA monitoring is a highly accurate method for early detection of asymptomatic metastatic recurrence in patients diagnosed with non-metastatic breast cancer and that ctDNA-based metastasis detection can precede symptoms and clinical detection by wide margins. These results provide the rationale for clinical trials in early breast cancer to test the clinical utility and benefit of ctDNA monitoring.

## Supporting Information

**Supplementary Methods**

**Review Process File**
