# Supplementary figures and images for: Serial monitoring of circulating tumor DNA in patients with primary breast cancer for detection of occult metastatic disease
Source: EMBO Mol Med. 2015 May 18;7(8):1034–47. doi: 10.15252/emmm.201404913 (PMC4551342; doi:10.15252/emmm.201404913)

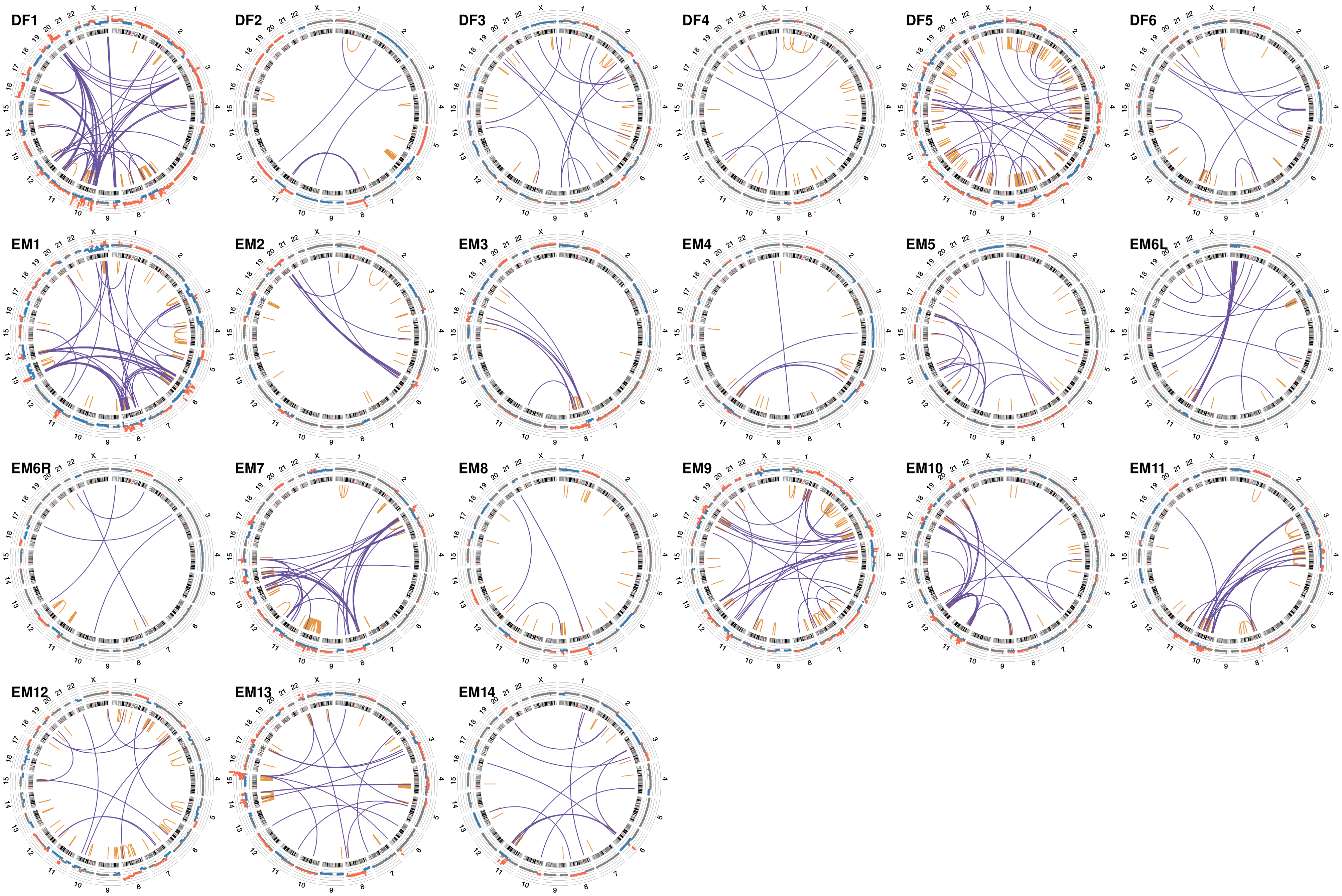

Supplement: Supplementary file 1 [file emmm0007-1034-sd1.gif]

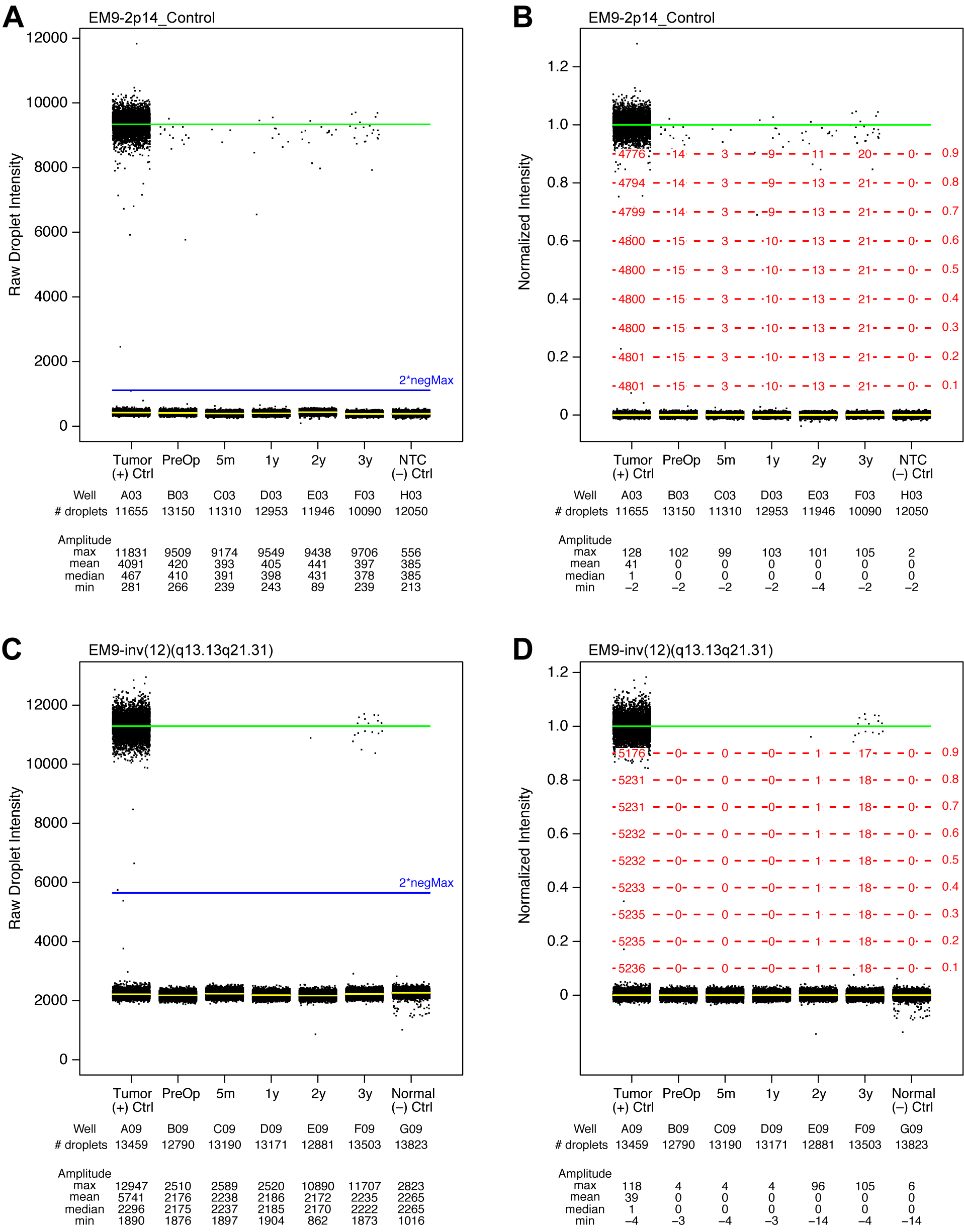

Supplement: Supplementary file 3 [file emmm0007-1034-sd3.gif]
