# Supplementary material for: Serial monitoring of circulating tumor DNA in patients with primary breast cancer for detection of occult metastatic disease
Source: EMBO Mol Med. 2015 May 18;7(8):1034–47. doi: 10.15252/emmm.201404913 (PMC4551342; doi:10.15252/emmm.201404913)
Supplement: Supplementary file 2 [file emmm0007-1034-sd2.pdf]

List of abbreviations

ANA	Anastrozole
BEV	Bevacizumab
CAP	Capecitabine
del	Chromosomal deletion
DOC	Docetaxel
DOX	Doxorubicin
EPI	Epirubicin
ER	Estrogen receptor
ERI	Eribulin
EXE	Exemestane
FEC	Fluorouracil + Epirubicin + Cyclophosphamide
FUL	Fulvestrant
HER2	Human epidermal growth factor receptor 2
inv	Chromosomal inversion
LET	Letrozole
M	Distant metastasis status at diagnosis (0, negative; 1, positive)
N	Positive lymph nodes at diagnosis (0, none; 1, 1–3; 2, 4–9; 3, >9)
NHG	Nottingham histological grade
PAC	Paclitaxel
PR	Progesterone receptor
RT	Radiotherapy
t	Chromosomal translocation
T	Tumor size (1, <=2cm; 2, 2–5cm; 3, >5cm)
TAM	Tamoxifen
TRA	Trastuzumab
VIN	Vinorelbine
y/o	Year old (at diagnosis)
ZOL	Zoledronate

Patient EM1 42 y/o

T2 (33 mm), N0, M0, NHG3, ER/PR pos, HER2 neg

ctDNA–detected recurrence 7 months prior to clinical recurrence

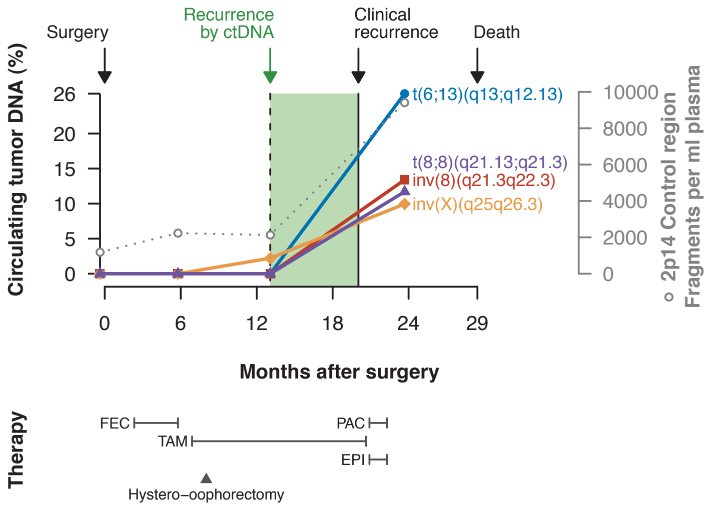

Patient EM2 57 y/o

T2 (28 mm), N1, M0, NHG2, ER/PR pos, HER2 neg

ctDNA–detected recurrence 16 months prior to clinical recurrence

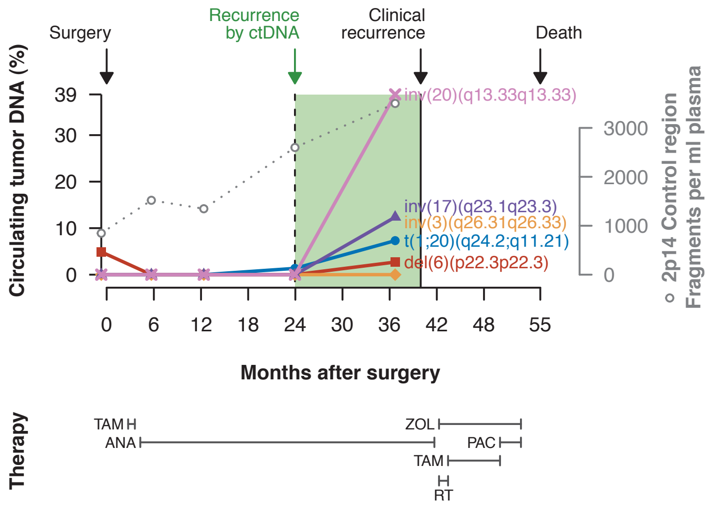

Patient EM3 78 y/o

T1 (20 mm), N2, M0, NHG3, ER/PR pos, HER2 neg

No ctDNA–detected recurrence despite clinical recurrence

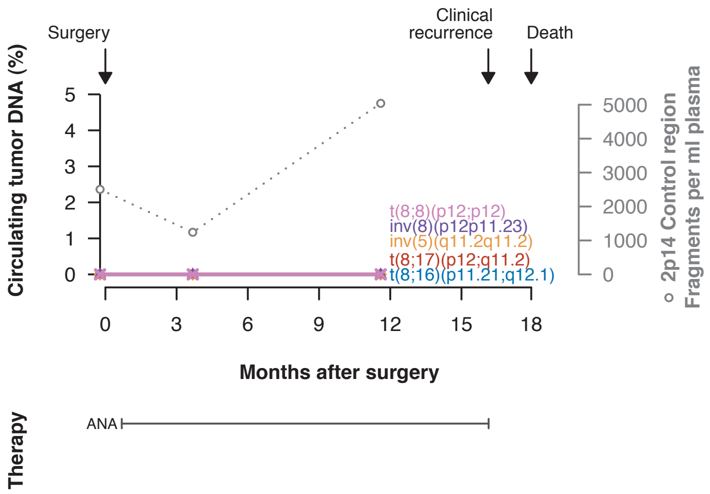

Patient EM4 34 y/o

T2 (28 mm), N0, M0, NHG2, ER/PR pos, HER2 neg

ctDNA–detected recurrence 4 months after clinical recurrence

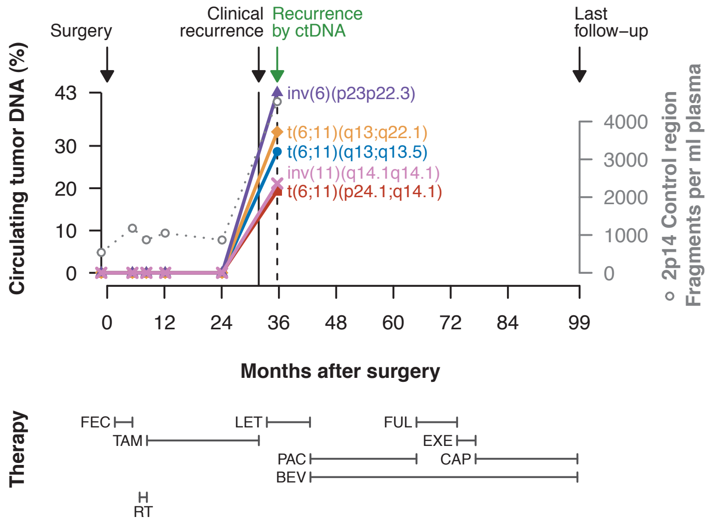

Patient EM5 61 y/o

T1 (12 mm), N0, M0, NHG1, ER/PR pos, HER2 neg

ctDNA–detected recurrence 36 months prior to clinical recurrence

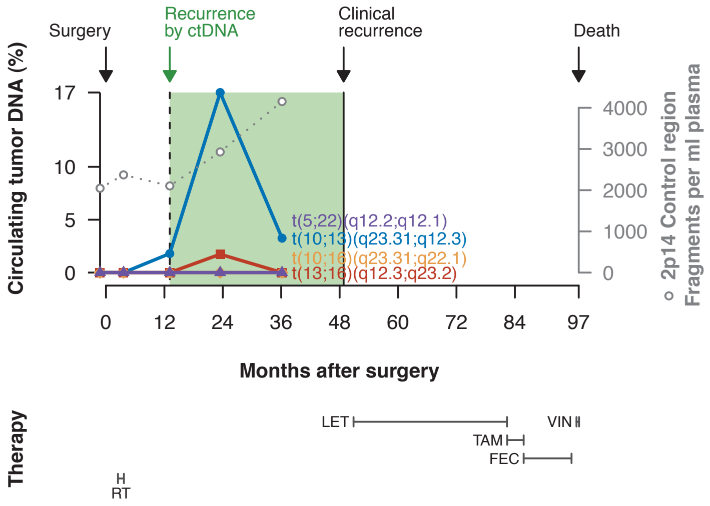

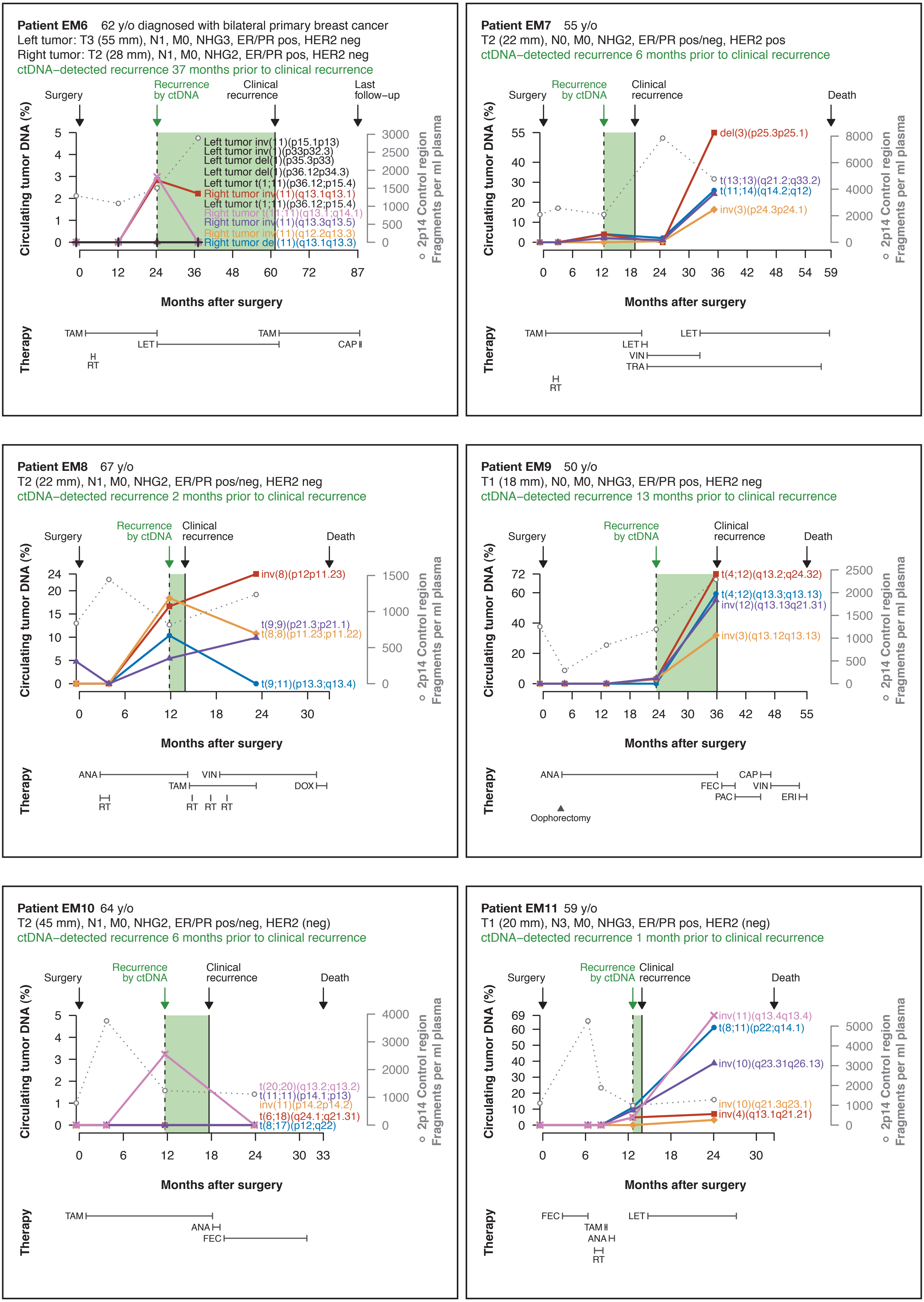

Patient EM6 62 y/o diagnosed with bilateral primary breast cancer
Left tumor: T3 (55 mm), N1, M0, NHG3, ER/PR pos, HER2 neg
Right tumor: T2 (28 mm), N1, M0, NHG2, ER/PR pos, HER2 neg
ctDNA–detected recurrence 37 months prior to clinical recurrence
Surgery
Recurrence by ctDNA
Clinical recurrence
Last follow–up
Left tumor inv(11)(p15.1p13)
Left tumor inv(11)(p33p32.3)
Left tumor del(1)(p35.3p33)
Left tumor del(1)(p36.12p34.3)
Left tumor t(1;11)(p36.12;p15.4)
Right tumor inv(11)(q13.1q13.1)
Left tumor t(1;11)(p36.12;p15.4)
Right tumor t(11;11)(q13.1;q14.1)
Right tumor inv(11)(q13.3q13.5)
Right tumor inv(11)(q12.2q13.3)
Right tumor del(11)(q13.1q13.3)
Circulating tumor DNA (%)
○ 2p14 Control region Fragments per ml plasma
Months after surgery
Therapy
TAM
LET
TAM
CAP
RT
Patient EM7 55 y/o
T2 (22 mm), N0, M0, NHG2, ER/PR pos/neg, HER2 pos
ctDNA–detected recurrence 6 months prior to clinical recurrence
Surgery
Recurrence by ctDNA
Clinical recurrence
Death
del(3)(p25.3p25.1)
t(13;13)(q21.2;q33.2)
t(11;14)(q14.2;q12)
inv(3)(p24.3p24.1)
Circulating tumor DNA (%)
○ 2p14 Control region Fragments per ml plasma
Months after surgery
Therapy
TAM
LET
LET
VIN
TRA
RT
Patient EM8 67 y/o
T2 (22 mm), N1, M0, NHG2, ER/PR pos/neg, HER2 neg
ctDNA–detected recurrence 2 months prior to clinical recurrence
Surgery
Recurrence by ctDNA
Clinical recurrence
Death
inv(8)(p12p11.23)
t(9;9)(p21.3;p21.1)
t(8;8)(p11.23;p11.22)
t(9;11)(p13.3;q13.4)
Circulating tumor DNA (%)
○ 2p14 Control region Fragments per ml plasma
Months after surgery
Therapy
ANA
VIN
TAM
DOX
RT
RT RT RT
Patient EM9 50 y/o
T1 (18 mm), N0, M0, NHG3, ER/PR pos, HER2 neg
ctDNA–detected recurrence 13 months prior to clinical recurrence
Surgery
Recurrence by ctDNA
Clinical recurrence
Death
t(4;12)(q13.2;q24.32)
t(4;12)(q13.3;q13.13)
inv(12)(q13.13q21.31)
inv(3)(q13.12q13.13)
Circulating tumor DNA (%)
○ 2p14 Control region Fragments per ml plasma
Months after surgery
Therapy
ANA
CAP
FEC
VIN
PAC
ERI
Oophorectomy
Patient EM10 64 y/o
T2 (45 mm), N1, M0, NHG2, ER/PR pos/neg, HER2 (neg)
ctDNA–detected recurrence 6 months prior to clinical recurrence
Surgery
Recurrence by ctDNA
Clinical recurrence
Death
t(20;20)(q13.2;q13.2)
t(11;11)(p14.1;p13)
inv(11)(p14.2p14.2)
t(6;18)(q24.1;q21.31)
t(8;17)(p12;q22)
Circulating tumor DNA (%)
○ 2p14 Control region Fragments per ml plasma
Months after surgery
Therapy
TAM
ANA
FEC
Patient EM11 59 y/o
T1 (20 mm), N3, M0, NHG3, ER/PR pos, HER2 (neg)
ctDNA–detected recurrence 1 month prior to clinical recurrence
Surgery
Recurrence by ctDNA
Clinical recurrence
Death
inv(11)(q13.4q13.4)
t(8;11)(p22;q14.1)
inv(10)(q23.31q26.13)
inv(10)(q21.3q23.1)
inv(4)(q13.1q21.21)
Circulating tumor DNA (%)
○ 2p14 Control region Fragments per ml plasma
Months after surgery
Therapy
FEC
LET
TAM
ANA
RT

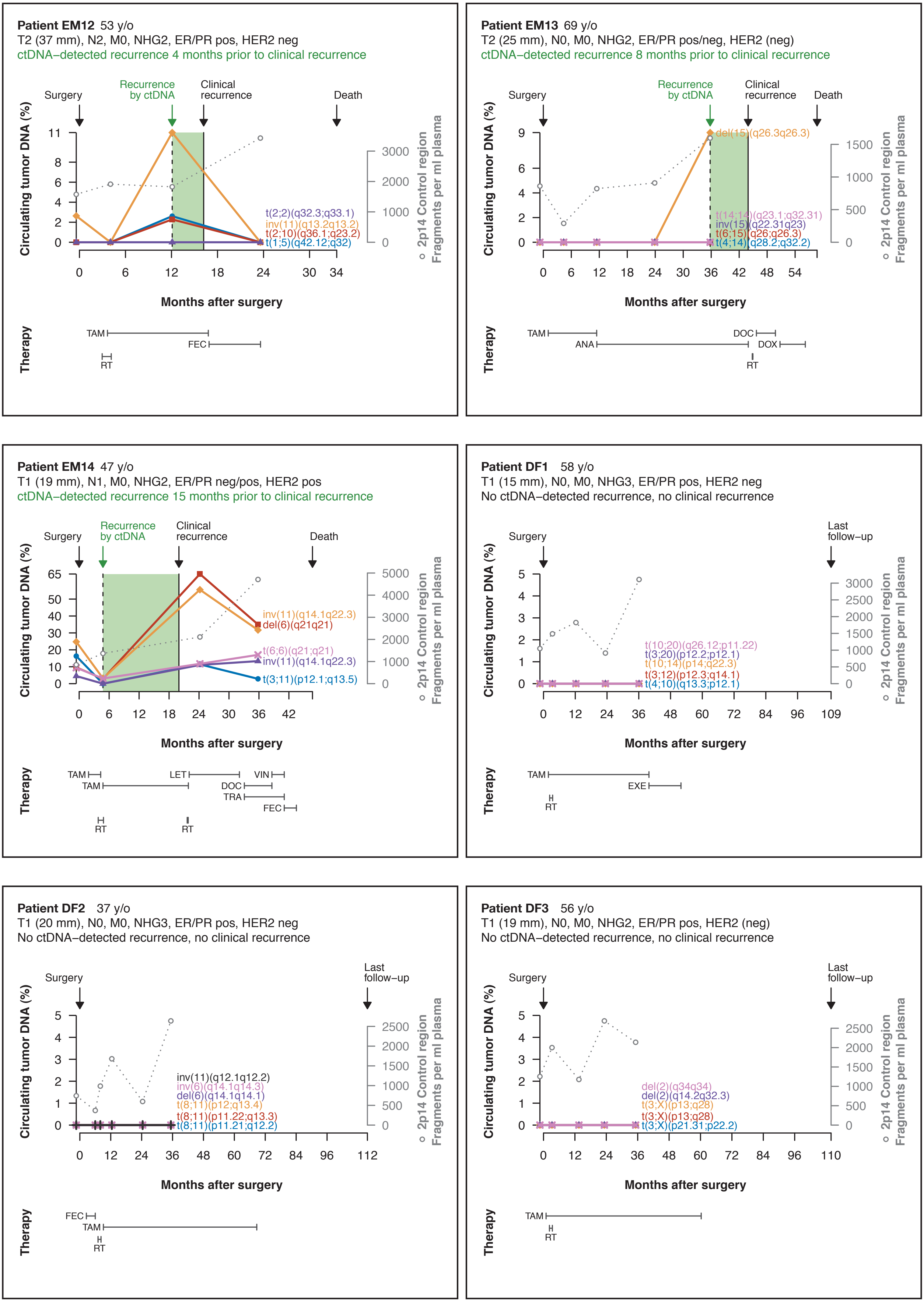

Patient EM12 53 y/o
T2 (37 mm), N2, M0, NHG2, ER/PR pos, HER2 neg
ctDNA−detected recurrence 4 months prior to clinical recurrence
Surgery
Recurrence by ctDNA
Clinical recurrence
Death
Circulating tumor DNA (%)
2p14 Control region Fragments per ml plasma
t(2;2)(q32.3;q33.1)
inv(11)(q13.2q13.2)
t(2;10)(q36.1;q23.2)
t(1;5)(q42.12;q32)
Months after surgery
Therapy
TAM
FEC
RT
Patient EM13 69 y/o
T2 (25 mm), N0, M0, NHG2, ER/PR pos/neg, HER2 (neg)
ctDNA−detected recurrence 8 months prior to clinical recurrence
Surgery
Recurrence by ctDNA
Clinical recurrence
Death
del(15)(q26.3q26.3)
t(14;14)(q23.1;q32.31)
inv(15)(q22.31q23)
t(6;15)(q26;q26.3)
t(4;14)(q28.2;q32.2)
Months after surgery
Therapy
TAM
ANA
DOC
DOX
RT
Patient EM14 47 y/o
T1 (19 mm), N1, M0, NHG2, ER/PR neg/pos, HER2 pos
ctDNA−detected recurrence 15 months prior to clinical recurrence
Surgery
Recurrence by ctDNA
Clinical recurrence
Death
inv(11)(q14.1q22.3)
del(6)(q21q21)
t(6;6)(q21;q21)
inv(11)(q14.1q22.3)
t(3;11)(p12.1;q13.5)
Months after surgery
Therapy
TAM
TAM
LET
VIN
DOC
TRA
FEC
RT
RT
Patient DF1 58 y/o
T1 (15 mm), N0, M0, NHG3, ER/PR pos, HER2 neg
No ctDNA−detected recurrence, no clinical recurrence
Surgery
Last follow−up
t(10;20)(q26.12;p11.22)
t(3;20)(p12.2;p12.1)
t(10;14)(p14;q22.3)
t(3;12)(p12.3;q14.1)
t(4;10)(q13.3;p12.1)
Months after surgery
Therapy
TAM
EXE
RT
Patient DF2 37 y/o
T1 (20 mm), N0, M0, NHG3, ER/PR pos, HER2 neg
No ctDNA−detected recurrence, no clinical recurrence
Surgery
Last follow−up
inv(11)(q12.1q12.2)
inv(6)(q14.1q14.3)
del(6)(q14.1q14.1)
t(8;11)(p12;q13.4)
t(8;11)(p11.22;q13.3)
t(8;11)(p11.21;q12.2)
Months after surgery
Therapy
FEC
TAM
RT
Patient DF3 56 y/o
T1 (19 mm), N0, M0, NHG2, ER/PR pos, HER2 (neg)
No ctDNA−detected recurrence, no clinical recurrence
Surgery
Last follow−up
del(2)(q34q34)
del(2)(q14.2q32.3)
t(3;X)(p13;q28)
t(3;X)(p13;q28)
t(3;X)(p21.31;p22.2)
Months after surgery
Therapy
TAM
RT

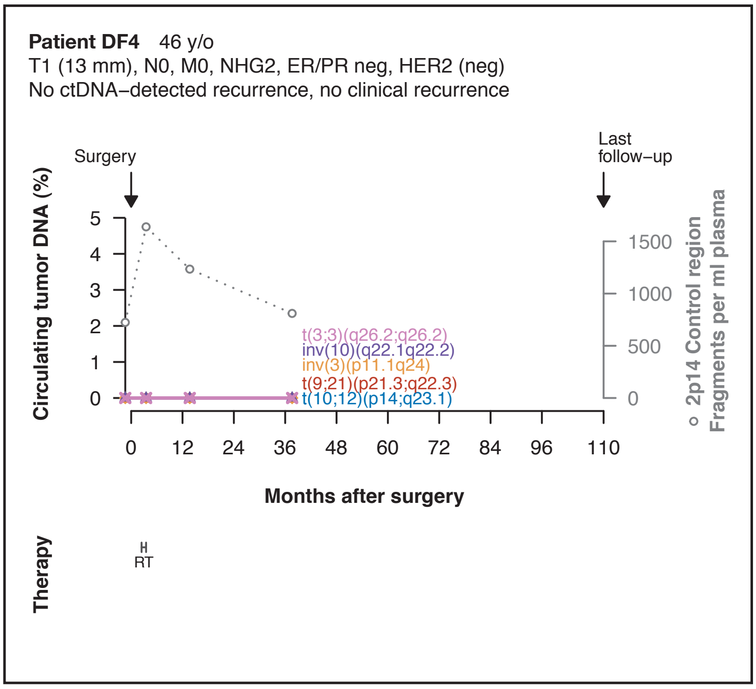
Patient DF4 46 y/o
T1 (13 mm), N0, M0, NHG2, ER/PR neg, HER2 (neg)
No ctDNA–detected recurrence, no clinical recurrence
Surgery
Last follow–up
Circulating tumor DNA (%)
5
4
3
2
1
0
1500
1000
500
0
2p14 Control region Fragments per ml plasma
t(3;3)(q26.2;q26.2)
inv(10)(q22.1q22.2)
inv(3)(p11.1q24)
t(9;21)(p21.3;q22.3)
t(10;12)(p14;q23.1)
0 12 24 36 48 60 72 84 96 110
Months after surgery
Therapy
RT

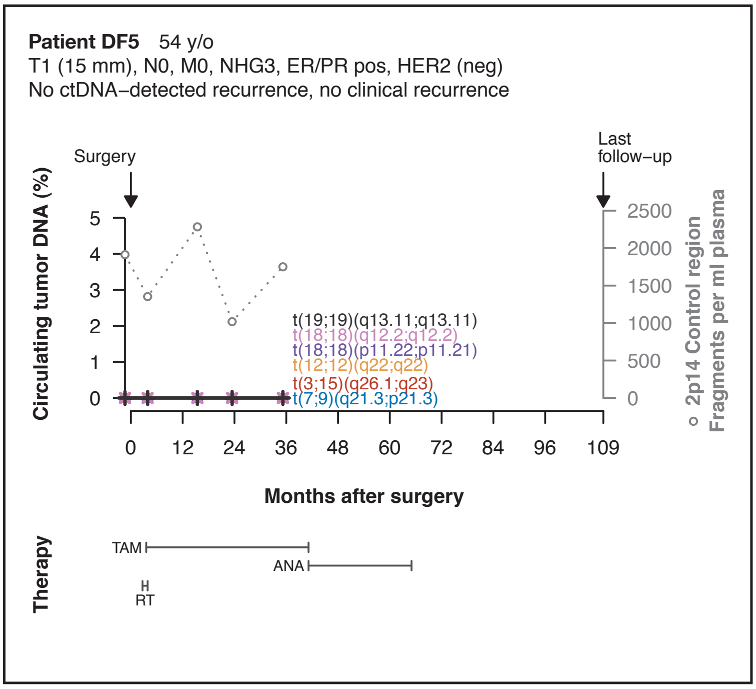
Patient DF5 54 y/o
T1 (15 mm), N0, M0, NHG3, ER/PR pos, HER2 (neg)
No ctDNA–detected recurrence, no clinical recurrence
Surgery
Last follow–up
Circulating tumor DNA (%)
5
4
3
2
1
0
2500
2000
1500
1000
500
0
2p14 Control region Fragments per ml plasma
t(19;19)(q13.11;q13.11)
t(18;18)(q12.2;q12.2)
t(18;18)(p11.22;p11.21)
t(12;12)(q22;q22)
t(3;15)(q26.1;q23)
t(7;9)(q21.3;p21.3)
0 12 24 36 48 60 72 84 96 109
Months after surgery
Therapy
TAM
ANA
RT

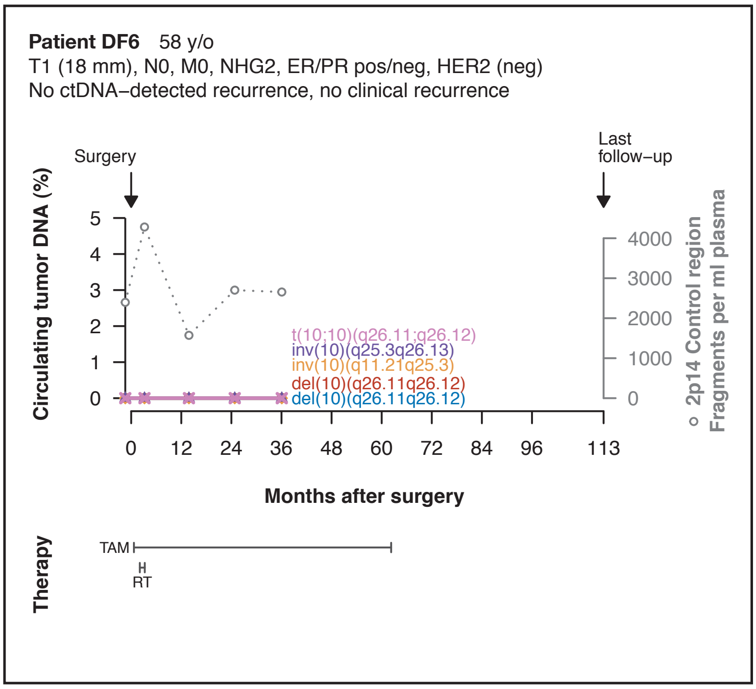
Patient DF6 58 y/o
T1 (18 mm), N0, M0, NHG2, ER/PR pos/neg, HER2 (neg)
No ctDNA–detected recurrence, no clinical recurrence
Surgery
Last follow–up
Circulating tumor DNA (%)
5
4
3
2
1
0
4000
3000
2000
1000
0
2p14 Control region Fragments per ml plasma
t(10;10)(q26.11;q26.12)
inv(10)(q25.3q26.13)
inv(10)(q11.21q25.3)
del(10)(q26.11q26.12)
del(10)(q26.11q26.12)
0 12 24 36 48 60 72 84 96 113
Months after surgery
Therapy
TAM
RT
