# Supplementary material for: Serial monitoring of circulating tumor DNA in patients with primary breast cancer for detection of occult metastatic disease
Source: EMBO Mol Med. 2015 May 18;7(8):1034–47. doi: 10.15252/emmm.201404913 (PMC4551342; doi:10.15252/emmm.201404913)
Supplement: Supplementary file 11 [file emmm0007-1034-sd11.docx]

**SUPPLEMENTARY LEGENDS**

## Figure S1 - Genomic chromosomal rearrangements and copy number aberrations for all patient tumors.

## Each plot represents the primary tumor genome of a patient characterized by whole-genome sequencing. For patient EM6, who presented with a bilateral primary breast cancer, the L or R suffix denotes left- or right-side tumor. Arcs inside the plot represent chromosomal rearrangements connecting two distinct loci in the tumor genome, with inter-chromosomal rearrangements shown in blue, and intra-chromosomal rearrangements shown in orange. The tumor copy number profile is plotted on the outside circle with gains in red, loss in blue, and normal copy number in grey. Plots were generated using Circos ([Krzywinski *et al*, 2009](#_ENREF_7)).

**Figure S2 - Circulating tumor DNA time-series and clinical course for all patients.**

Each patient plot shows four to six chromosomal rearrangements found in the primary patient tumor and quantified in blood plasma pre-surgically and at several time-points after surgery. The fraction of the rearrangements relative to total circulating tumor DNA (ctDNA) in each plasma sample are shown as colored points connected with colored lines and are labeled with the type of rearrangement (t, inv, or del; see list of abbreviations in this Figure) followed by chromosome numbers and chromosome regions involved (chromosome arm and band). The quantity of total circulating DNA as estimated by the control 2p14 assay is shown by the grey open circle markers connected by dashed lines (units indicated on the right-side Y-axis). The recurrence by ctDNA time-point is defined as the earliest follow-up plasma sample with ctDNA from at least one rearrangement detected at a level greater than 0%. In patients with clinical recurrence which had a recurrence by ctDNA detected earlier, the time-gain is highlighted with a green rectangle. Clinical data are from patient and pathology records. If not assessed clinically, HER2 status is presented in parentheses and was derived from copy number analysis of the whole-genome sequencing data.

## Figure S3 - Droplet digital PCR data normalization.

## To enable an unbiased, uniform, and operator-blinded automatic thresholding of ddPCR data, droplet fluorescent intensity measurements of each assay across samples were normalized to a relative scale ranging from 0 to 1 by scaling to the negative control (- Ctrl) and positive control (+ Ctrl) droplet intensities, for each assay, using custom scripts (see Supplementary Methods). To illustrate the transformation steps, example (A, D) unprocessed and (B, D) normalized intensity data (Y-axis) are shown for (A, B) 2p14 control assay and (C, D) inv(12)(q13.13q21.31) assay for patient EM9 (one of the replicates), across input samples (X-axis). In (A, D), the blue line (“2*negMax”) is drawn for a given ddPCR assay at 2-times the maximum droplet intensity of the negative control (no-template control, NTC; or matched normal DNA, Normal) reaction well, defining the data from the given assay into an upper and lower portion. Yellow lines are drawn at the median value for the droplets in the lower portion within each well (“lowerMed”), and the green line is drawn across at the median value for the upper portion of the positive control primary tumor DNA well (“posUpperMed”). Data are then scaled to 0 and 1 (B, D) corresponding to these lowerMed and posUpperMed values, respectively. The red dashed line is drawn for 9 example threshold values, from 0.1 to 0.9, and numbers given within the lines indicate the count of droplets above the threshold for the respective well. Summary statistics for each well is provided below each plot.

**Table S1.** Sequencing statistics and numbers of rearrangements.

**Table S2.** Chromosomal rearrangements identified in primary tumors.

**Table S3.** Selected chromosomal rearrangements as personalized ctDNA biomarkers.

**Table S4.** Summary of somatic validation and personalized assay validation.

**Table S5.** Rearrangement and 2p14 region measured quantities in plasma.

**Table S6.** Patient ctDNA-based recurrence detection lead times.

**Table S7.** Logistic regression analysis of ctDNA as a predictive factor.
