# Supplementary material for: Serial monitoring of circulating tumor DNA in patients with primary breast cancer for detection of occult metastatic disease
Source: EMBO Mol Med. 2015 May 18;7(8):1034–47. doi: 10.15252/emmm.201404913 (PMC4551342; doi:10.15252/emmm.201404913)
Supplement: Supplementary file 12 [file emmm0007-1034-sd12.pdf]

# Serial Monitoring of Circulating Tumor DNA in Patients with Primary Breast Cancer for Detection of Occult Metastatic Disease

Eleonor Olsson, Christof Winter, Anthony George, Yilun Chen, Jillian Howlin, Man-Hung Eric Tang, Malin Dahlgren, Ralph Schulz, Dorthe Grabau, Danielle van Westen, Mårten Fernö, Christian Ingvar, Carsten Rose, Pär-Ola Bendahl, Lisa Rydén, Åke Borg, Sofia K. Gruvberger-Saal, Helena Jernström, and Lao H. Saal

## SUPPLEMENTARY INFORMATION

## TABLE OF CONTENTS

METHODS ........ 2
- Extraction of genomic DNA ........ 2
- Isolation of circulating DNA from plasma ........ 2
- Sample preparation for sequencing ........ 2
- Cluster generation and sequencing ........ 3
- Alignment of sequencing paired-end reads ........ 3
- *In silico* identification of putative chromosomal rearrangements ........ 3
- Copy number analysis ........ 4
- Annotation of rearrangements ........ 4
- Filtering rearrangement calls ........ 4
- Reconstruction of exact breakpoint fusion sequence ........ 4
- Rearrangement nomenclature ........ 5
- Primer and probe design ........ 6
- PCR validation of structural variants ........ 6
- Droplet digital PCR ........ 6
- Droplet digital PCR data normalization ........ 7
- Calculation of template DNA concentration and ctDNA percentage ........ 8
- Receiver operating characteristic (ROC) curve analysis ........ 8
- Statistical Analyses ........ 9
- Logistic regression analysis with ctDNA level as covariate ........ 9
- Univariable logistic regression analysis with clinical covariates ........ 10
- Software implementation ........ 11

SUPPLEMENTARY REFERENCES ........ 11

## METHODS

### Extraction of genomic DNA

For tumor DNA extraction, approximately 30 mg of frozen tumor specimen was lysed in RLT Plus buffer and immediately homogenized for 2 x 4 minutes at 30 Hz using a TissueLyser (Qiagen). The AllPrep DNA/RNA kit (Qiagen) was used to isolate the nucleic acids. The procedure was automated on a Qiacube extraction robot (Qiagen). For all 20 patients, normal genomic DNA was isolated from 1 mL of blood (1-year time-point for all patients except patient EM14 for which a 5-month time-point was used) using the Wizard Genomic DNA Purification method (Promega) according to manufacturer's instructions.

### Isolation of circulating DNA from plasma

Within two hours of collection, the patient EDTA blood samples were centrifuged at 1200g for 10 minutes at room temperature (RT) to separate the plasma, white blood cell, and red blood cell fractions. Aliquots of each fraction were stored at -80 °C. The circulating DNA present in the blood plasma was isolated using the QIAamp UltraSens Virus Kit (Qiagen). DNA LoBind tubes (Eppendorf) were used wherever possible. Briefly, blood plasma was thawed on wet ice and then centrifuged at 10000g for 10 minutes at 4 °C and 0.5 mL of the supernatant was transferred to a new tube for circulating DNA isolation according to manufacturer's instructions with some modifications. The samples were eluted in 100 µl Buffer EB.

### Sample preparation for sequencing

For the 20 patients, the genomic DNA for 21 primary tumors was sequenced (patient EM6 had bilateral breast cancer). In addition, in order to filter germline and false-positive rearrangements, normal DNA samples were sequenced for 3 of these patients as well as normal DNA samples from 7 non-matched individuals. The tumor and normal DNA samples were measured by a ND-1000 NanoDrop spectrophotometer (NanoDrop Technologies), and 2.4 $\mu$g was sheared to 700 bp average length using the S220 Focused-Ultrasonicator Instrument (Covaris) with the following settings: duty cycle 5%, intensity 3, cycles per burst 200, time 30 s at 5 °C. After shearing the fragment sizes were determined using the 2100 Bioanalyzer (Agilent Technologies). Sample preparation was performed using the TruSeq DNA Sample Preparation Kit (Illumina) according to the TruSeq Sample preparation guide (Part # 15005180 Rev. A) using the low-throughput protocol. Briefly, 1 $\mu$g of sheared genomic DNA was used for each library preparation. Following end-repair and adenylation of 3'-ends, the samples were indexed with paired-end adaptors. After purification, each library was size separated on a 2% agarose gel (Invitrogen)

with empty lanes between all samples. Fragments between 550 bp and 950 bp were cut out and purified before PCR amplification. The size of each library was validated on the 2100 Bioanalyzer and the concentration was measured on Qubit (Invitrogen). Compatible libraries were pooled prior to cluster generation.

### Cluster generation and sequencing

Sequencing clusters were generated on a cBot instrument (Illumina) using TruSeq Cluster Kit V2 cBot HS (Illumina) and v1.5 or v3 PE Flow Cells (Illumina). Paired-end sequencing of 2x50 bp, 2x100 bp, or 2x150 bp plus index was performed in-house on a HiSeq 2000 or HiSeq 2500 sequencer using TruSeq SBS Kit v3 HS chemistry (Illumina) according to manufacturer's instructions.

### Alignment of sequencing paired-end reads

Paired-end reads were aligned to Genome Reference Consortium Human Build 37 (GRCh37; used in the 1000 genomes project phase 1; downloaded from ftp://ftp.1000genomes.ebi.ac.uk/vol1/ftp/technical/reference/human_g1k_v37.fasta.gz). Alignments were performed using Novoalign v2.07.18 (Novocraft Technologies) with soft-clipped read alignments (option -o Softclip). Data from samples run on multiple lanes of a flow cell were merged into one BAM file per sample using Novosort V1.00.01 (Novocraft Technologies). Duplicate read-pairs in the BAM files were flagged with Picard MarkDuplicates v1.66 and ignored in subsequent analyses. Sequencing statistics are presented in Supplementary Table S1.

### *In silico* identification of putative chromosomal rearrangements

To identify chromosomal rearrangements, aligned tumor BAM files were first searched with BreakDancer (Chen *et al*, 2009) using default options for discordant read-pairs, resulting in the prediction of translocations, duplications, inversions, and deletions (BreakDancer types CTX, ITX, INV, and DEL, respectively). To reduce false-positive rearrangement predictions due to misalignment of paralogous sequences, all rearrangement-supporting discordant read-pairs were re-aligned to the reference genome using Novoalign with the 1000 top-scoring alignments above a moderate alignment score being reported (parameters -r Exhaustive 1000 -t 250). Initially discordant read-pairs that became concordant after this exhaustive re-alignment step were discarded.

**Copy number analysis**

Using whole-genome sequencing data, copy number was determined across the genome in windows of 50 kb using FREEC version 5.6 with default parameters (Boeva *et al*, 2011).

**Annotation of rearrangements**

Identified rearrangement breakpoint ends were annotated with RefSeq genes, sequence gaps (gaps track), and repetitive elements (RepeatMasker track) for the human reference genome (hg19), all obtained from the UCSC Table Browser (http://genome.ucsc.edu/cgi-bin/hgTables), and with entries from the Database of Genomic Variants (http://dgv.tcag.ca/). Further, each rearrangement was annotated with a list of matching rearrangements in the other tumor samples, and in 10 normal DNA samples (3 matched normals, and 7 additional unmatched normals with similar sequencing depth).

**Filtering rearrangement calls**

To deplete the list of predicted chromosomal rearrangements for potential non-specific rearrangements and false-positives before experimental validation, the following filtering criteria were applied:

1. At least 2 discordant read-pairs supporting the rearrangement.
2. No satellite DNA (RepeatMasker class "Satellite") present within 1 kb of any of the two breakpoint ends of the rearrangement.
3. No sequence gap (UCSC track "gaps") present within 1 kb of any of the two breakpoint ends.
4. No matching rearrangement in other tumor samples within 1 kb (both breakpoint ends matched).
5. No matching rearrangement in other normal samples within 1 kb (both breakpoint ends matched).
6. For intra-chromosomal rearrangements, size of the rearrangement (distance between the two breakpoint ends) greater than 1 kb.
7. Both breakpoint ends on chromosomes 1–22 or X (i.e. no involvement of non-standard sequence contigs present in the human genome reference).

**Reconstruction of exact breakpoint fusion sequence**

To facilitate detecting short fragments (<200 bp) of rearranged DNA in blood plasma by PCR, identifying the exact sequence of the breakpoints and a tight primer design is crucial. To this end, we developed SplitSeq, a computational pipeline that can reconstruct the exact fusion sequence

spanning chromosomal rearrangements. A discrete rearrangement consists of two breakpoint genomic positions that are fused and made contiguous. Briefly, SplitSeq proceeds as follows. Using discordant read-pairs as anchors, the regions around the breakpoints are searched for split-reads with at least 2 soft-clipped bases (of base quality ≥15) that do not match the reference (soft-clipping in Novoalign can occur either due to mismatches or due to low base qualities at any end of a read). The same regions are also searched for reads with unmapped mates (i.e. read-pairs where only one read of the pair is aligned), and alignment of the unmapped mates to the breakpoint regions is attempted. Alignment is done with a reduced gap extension penalty (parameter -x 1) starting with the full read length, followed by two rounds of trimming the read to 2/3 of its previous length on either end. Any mapped split-read identified with this procedure with at least 2 soft-clipped bases that do not match the reference is added to the list of split reads. Next, all soft-clipped reads from this list are sorted by their clipping position in the reference genome in order to identify putative exact breakpoint positions. Reference genome positions with support of less than a total of 6 clipped bases are discarded. For all other positions, starting with those with the highest number of clipped-base support, all pairs connecting the two breakpoints (defined by the discordant read-pair anchors) are searched for perfect matches of all clipped bases when aligned to a fusion sequence reconstructed from the two breakpoint positions. Reconstructed fusion sequences where both breakpoints have clipped-base support on both sides (i.e. left of and right of each breakpoint position) are chosen first. If no such reconstructed cases exist, cases where only one breakpoint position has clipped-base support on both sides are chosen. If no such cases exist, cases where both breakpoints have clipped-base support on only one side are selected. If no such cases exist, no exact breakpoint positions are called, and no exact fusion sequence is reconstructed.

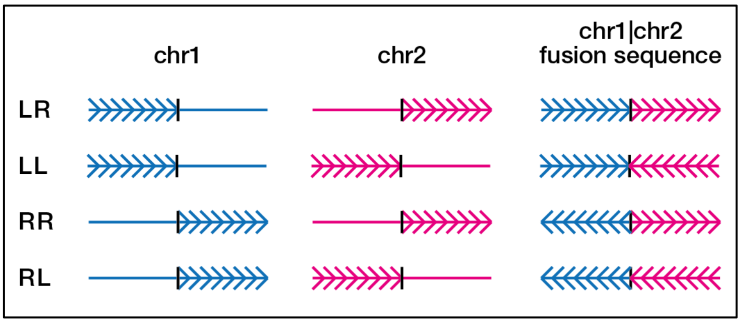


**Rearrangement nomenclature**

To unambiguously describe a rearrangement such that its exact fusion sequence can be reconstructed, the chromosome and position of both breakpoints plus the information on whether the DNA left of or right of each breakpoint is involved in the fusion event is needed. Here were use the following nomenclature: for each breakpoint, L denotes that the genomic DNA left of the breakpoint when viewed in a genome browser (i.e. towards lower genomic coordinates) participates in the fusion, whereas R denotes that the genomic DNA right of the breakpoint when viewed in a genome browser (i.e. towards higher genomic coordinates) participates in the fusion (see drawing above). We found that the

L/R notation is more intuitive and creates less confusion than the use of strandedness (plus or minus) or strand orientation (5’ or 3’). All chromosomal rearrangements used in the study are summarized in Supplementary Table S1, listed in Supplementary Table S2, and shown in Circos plots (Krzywinski *et al*, 2009) in Supplementary Fig. S1.

**Primer and probe design**

Quantitative PCR assays for droplet digital PCR, spanning the predicted breakpoints of selected structural variants, were manually designed by using the Primer Express 3.0.1 software (Applied Biosystems). Traditional guidelines for design of TaqMan probes and primers were followed (Applied Biosystems User Guide). The size of amplicons were designed to be as short as possible due to the highly fragmented nature of circulating DNA. The primers and probe were generally placed >1 bp from the predicted breakpoint (in either direction). The probes were labeled with FAM and quenched by an internal ZEN and a 3’-IBFQ molecule. The assay for an invariant region of chromosome 2p14 was designed similarly. All oligonucleotides were ordered from Integrated DNA Technologies (IDT) and the sequences are provided in Supplementary Table S3.

**PCR validation of structural variants**

Touchdown PCR was used to validate the assays for the predicted breakpoints of the structural variants. To distinguish tumor-specific variants from germline variants, each primer pair PCR assay was performed using primary tumor DNA and matched normal DNA for each patient. PCR reactions were performed in a total volume of 10 μL with Phusion Master Mix 1x (Thermo Scientific), 250 nM of each primer, and 10 ng of template DNA. PCR cycling conditions were as follows: initial denaturation at 98 °C for 2 minutes followed by 11 cycles of 98 °C for 10 s, 70°C (–1 °C/cycle) for 30 s and 72 °C for 15 s and then 29 cycles of 98 °C for 10 s, 60 °C for 30 s and 72 °C for 15 s and a final elongation at 72 °C for 5 min. The PCR products were analyzed on a Caliper LabChip XT microcapillary gel instrument (PerkinElmer) for band size and specificity.

**Droplet digital PCR**

Structural variants that were confirmed to be somatic by touchdown PCR were also analyzed with droplet digital PCR (ddPCR) using the QX100 Droplet Digital PCR System (Bio-Rad). In ddPCR, sample DNA, rearrangement-specific primers, quenched fluorescent probe, and PCR mix are partitioned into thousands of uniform nanoliter-size droplet reactions. PCR is carried out, wherein only if one or more target molecule is present in the droplet and successfully amplified will the fluorescent probes be unquenched by polymerase-mediated hydrolysis. All assays, including both primers and probes, were tested both on primary tumor DNA and matched normal

blood before continuing with ctDNA isolated from blood plasma from the same patient. Because the random distribution of zero, one, or more than one target molecule into each droplet follows a Poisson distribution, the ratio of the number of positive droplets to number of total recorded droplets can be Poisson-corrected to derive a highly quantitative count of the absolute number of target molecules present in the input sample(Hindson *et al*, 2011). Droplet digital PCR reactions were performed in a total volume of 20 μL with Master Mix ddPCR mix (Bio-Rad), 250 nM of each primer, 250 nM of probe and 4 μL of template ctDNA. The cycling conditions for ddPCR were as follows: initial denaturation at 95 °C for 10 minutes followed by 10 cycles of 94 °C for 30 s, 65 °C (–0.7 °C/cycle) for 60 s and then 35 cycles of 94 °C for 30 s, 58 °C for 60 s and a final step at 98 °C for 10 min. The ramp rate between any two consecutive steps was 2.5 °C/s. Matched tumor DNA and normal DNA were utilized as positive and negative controls, respectively, for each ddPCR rearrangement assay, and tumor DNA and no-template control (water), respectively, as controls for the 2p14 assay. Droplets were read by the QX100 Droplet Reader. The results were evaluated with QuantaSoft version 1.4.0 (Bio-Rad). Because all experiments were run using assays with single-fluorophore probes, droplets that appeared as outliers in 2D amplitude clustering plots, with simultaneous outlier positive intensities in both fluorescent channels, were removed as these likely represent false-positive signals due to nanoparticulate contamination such as plastic (25 droplets were removed out of >1.9 million droplets analyzed).

**Droplet digital PCR data normalization**

Because the feature in QuantaSoft software for automatic thresholding of droplet intensities frequently failed, the droplet intensity values were exported from QuantaSoft in order to be able to apply an automatic, unbiased, reproducible, and operator independent thresholding method. Custom scripts were developed and used to import and normalize the intensity values in R (version 2.14.1; http://www.r-project.org). Normalizing the data facilitated employing one intensity threshold for all samples in order to define negative droplets (below threshold) and positive droplets (above threshold). Using centering and scaling, this procedure normalized each individual assay's droplet fluorescent intensity data to a new scale where 0 corresponds to no intensity (at the median intensity of negative control droplets) and 1 corresponds to full intensity (at the median intensity of positive control droplets) and was carried out as described below, and is illustrated in Supplementary Fig. S3. For each rearrangement assay:

1. Determine negMax = maximum intensity value in the negative control well.
2. For each ddPCR reaction well, define a lower group of droplets with intensity <= 2*negMax and an upper group of droplets with intensity > 2*negMax.

3. For each well, determine lowerMed = median intensity value of the lower group of droplets.
4. For each well, subtract lowerMed from each intensity value (bringing all medians to zero).
5. Determine posUpperMed = median intensity value in the upper group of droplets in the positive control well.
6. For each well, divide each intensity value by posUpperMed (setting the median of the upper droplets in the positive control to 1).
7. Set threshold t to 0.5 to define negative droplets (below threshold) and positive droplets (greater than or equal to threshold).

**Calculation of template DNA concentration and ctDNA percentage**

The number of fragments per μl input purified circulating DNA ($C_{Vi}$) was calculated from the number of positive droplets $P$, total number of droplets analyzed $T$, droplet volume $V_d$ ($0.91 \times 10^{-3}$ μl), ddPCR reaction volume $V_r$ (20 μl; includes PCR mix, primers, probe, input DNA), and volume (4 μl) of purified circulating DNA input into the reaction $V_i$, using the formula $C_{Vi} = (\frac{-\ln(1-\frac{P}{T})}{V_d})(\frac{V_r}{V_i})$. For each plasma sample, the concentration of a normal non-rearranged 132 bp region of chromosome 2p14 as well as of four to six tumor-specific rearrangements was determined. To control for possible variability in the efficiency of plasma DNA isolation or degradation of cell-free circulating DNA during long-term storage of plasma, for each rearrangement, ctDNA level was estimated as a percentage of total circulating DNA by dividing the quantity of measured rearrangement by the quantity of the 2p14 control region (see Supplementary Table S5). In the event that more than one extraction was performed, the ctDNA percentage was calculated within the relevant extraction, and for plotting, the average value was used (Supplementary Fig. S2).

**Receiver operating characteristic (ROC) curve analysis**

Because the sensitivity and specificity of ctDNA-based monitoring for occult disease can be influenced by the fluorescent intensity threshold used in calling ddPCR droplets positive or negative, we applied a ROC curve analysis. In this analysis, the threshold t (as defined above), for each assay, was incrementally varied from 0.0 to 1.0 in 0.1 increments and applied to the normalized data from all samples, defining negative droplets (below threshold) and positive droplets (above threshold). For each time-point, the concentration (in copies per nanoliter ddPCR reaction) of each rearrangement in blood plasma was then calculated as above for all patients, and

the rearrangement with the highest concentration was used to represent each time-point as this was thought to be most clinically relevant. Thus, ctDNA was represented and analyzed using a single covariate. If a patient had at least one detectable rearrangement ($C_{Vi} > 0$ copies/nl, i.e. ctDNA >0%) in a blood plasma sample at any time after surgery, the patient was predicted as having a recurrence (recurrence positive). Patients with $C_{Vi} = 0$ copies/nl for all rearrangements at all time-points after surgery were predicted as recurrence-free (recurrence negative). The predicted recurrence state was then compared with the known true recurrence state obtained from the clinical records in order to determine true positive (TP), true negative (TN), false positive (FP), and false negative predictions (FN). Sensitivity was calculated as TP/(TP+FN), and specificity was calculated as TN/(TN+FP). Sensitivity was plotted against 1–specificity for each threshold t, producing a ROC curve (Fig. 5a). At thresholds t from 0.35 to 0.95, identical sensitivity and specificity results were obtained.

**Statistical Analyses**

All statistical calculations were done in R version 2.14.1. Confidence intervals for sensitivity, specificity, and area under the ROC curve were calculated based on the Clopper-Pearson exact binomial distribution method using the R package *binom* v1.1-1 with the function call *binom.confint(x, n, conf.level=0.95, methods="exact")* representing x successes in n independent binomial trials. Input values were x=13 and n=14 for sensitivity, x=6 and n=6 for specificity, and x=19 and n=20 for area under the ROC curve. All P-values and confidence intervals calculated are two-sided except for the confidence interval for specificity (one-sided 95% confidence interval since the proportions are estimated to 1; *i.e.*, *conf.level=0.90* in the function call).

**Logistic regression analysis with ctDNA level as covariate**

All regression analyses were done using the R package *glm* and *brglm*. Follow-up ctDNA percentage levels measured in patient plasma (see Supplementary Fig. S2) were used as a continuous covariate in logistic regression analysis. At each time-point, the rearrangement with the maximal ctDNA percentage value was used as this was thought to be most clinically relevant. Only the most recent plasma sample time-point before an event was used, resulting in one ctDNA percentage level per patient. Standard logistic regression modeling (R package *glm* with family binomial and link logit), failed to converge for recurrence as dependent variable due to quasi-complete separation of ctDNA level between DF patients (Fig. 5D, lower black dots) and EM patients (Fig. 5D, upper black dots). We therefore employed Firth logistic regression (Firth, 1993) implemented in the R package *brglm* (Kosmidis, 2013), which uses a penalized maximum likelihood approach that allows reliable estimation also for separated data (Heinze, 2006). Since

we assumed that e.g. a 10-unit increase in ctDNA percentage from 0% to 10% may have a different prognostic implication than an increase of the same magnitude from 50% to 60%, we allowed for non-linear effects of ctDNA levels on the risk. To this end, we used fractional polynomials of degree 1, corresponding to a transformation $x^k$ with k taking the values {–2, –1, –0.5, 0, 0.5, 1, 2, 3}, with x being the ctDNA level in percent, and where k=0 was defined as a $\log_2$-transformation and k=1 corresponds to no transformation (linear effect). To avoid undefined values in the $\log_2(x)$-transformation or the $x^k$ transformation for $k \leq 0$, we added 0.1% to the ctDNA value before these transformations (e.g., for k=0, $\log_2(x+0.1)$). For each k, we fitted one model with $(ctDNA)^k$ as covariate and death as dependent variable (Table 1), and one model with $(ctDNA)^k$ as covariate and clinical recurrence as dependent variable (Supplementary Table S6). We then used the Akaike information criterion (AIC) to select the best transformation by summing the AIC for the two models (death and clinical recurrence) for each of the eight transformations, and selecting the transformation with the minimal summed AIC. The best models were obtained using a $\log_2$-transformation, followed by the square-root (sqrt) transformation (k=0.5), with the AIC being 39.05 and 39.38 for $\log_2$ and sqrt, respectively, making the models using $\log_2$-transformed ctDNA levels 1.18 times as probable to minimize the information loss as the sqrt-based models (exp((39.38 – 39.05)/2) = 1.18). AIC for the untransformed (k=1) models was 40.38, making them 1.94 times less likely to minimize information loss as the $\log_2$-transformed models. Since the input ctDNA level is $\log_2$-transformed, the resulting odds ratios are for an increased risk per unit increase in $\log_2$ (each unit corresponding to a 2-fold increase in percentage ctDNA; e.g. from 1% to 2%, or 3% to 6%).

**Univariable logistic regression analysis with clinical covariates**

The primary diagnosis clinical parameters of tumor size (T3, >5 cm, *versus* T1, ≤2 cm, and T2, 2-5 cm), number of positive lymph nodes (N1, 1-3 positive, N2, 4-9 positive, and N3 >9 positive nodes *versus* N0, none), Nottingham histological grade (G3 *versus* G1 and G2), estrogen receptor status (ER-negative *versus* ER-positive), progesterone receptor status (PR-negative *versus* PR-positive), and HER2 status (HER2-positive *versus* HER2-negative), and the Nottingham Prognostic Index (NPI; calculated as 0.2 * S [size of index tumor in cm] + N [number of nodes; 0=1, 1-2=2, ≥3=3] + G [G1=1, G2=2, G3=3]) were each used as a single covariate in univariable Firth logistic regression analyses as described in the previous section. For patient EM6 with bilateral breast cancer, the left-side tumor with worse clinical prognostic features was used (Table 1). Clinical variables were coded dichotomized as such because there was only one T3 class case, one G1 case, and one N3 case.

### Software implementation

Python, R, and Bash shell scripts were written to run all bioinformatics analyses.

## SUPPLEMENTARY REFERENCES


Boeva V, Zinovyev A, Bleakley K, Vert JP, Janoueix-Lerosey I, Delattre O, Barillot E (2011) Control-free calling of copy number alterations in deep-sequencing data using GC-content normalization. *Bioinformatics* **27:** 268-269

Chen K, Wallis JW, McLellan MD, Larson DE, Kalicki JM, Pohl CS, McGrath SD, Wendl MC, Zhang Q, Locke DP, Shi X, Fulton RS, Ley TJ, Wilson RK, Ding L, Mardis ER (2009) BreakDancer: an algorithm for high-resolution mapping of genomic structural variation. *Nat Methods* **6:** 677-681

Firth D (1993) Bias reduction of maximum likelihood estimates. *Biometrika* **80:** 27-38

Heinze G (2006) A comparative investigation of methods for logistic regression with separated or nearly separated data. *Stat Med* **25:** 4216-4226

Hindson BJ, Ness KD, Masquelier DA, Belgrader P, Heredia NJ, Makarewicz AJ, Bright IJ, Lucero MY, Hiddessen AL, Legler TC, Kitano TK, Hodel MR, Petersen JF, Wyatt PW, Steenblock ER, Shah PH, Bousse LJ, Troup CB, Mellen JC, Wittmann DK, Erndt NG, Cauley TH, Koehler RT, So AP, Dube S, Rose KA, Montesclaros L, Wang S, Stumbo DP, Hodges SP, Romine S, Milanovich FP, White HE, Regan JF, Karlin-Neumann GA, Hindson CM, Saxonov S, Colston BW (2011) High-throughput droplet digital PCR system for absolute quantitation of DNA copy number. *Anal Chem* **83:** 8604-8610

Kosmidis I. (2013) brglm: Bias reduction in binary-response Generalized Linear Models.

Krzywinski M, Schein J, Birol I, Connors J, Gascoyne R, Horsman D, Jones SJ, Marra MA (2009) Circos: an information aesthetic for comparative genomics. *Genome Res* **19:** 1639-1645
