# Supplementary material for: Serial monitoring of circulating tumor DNA in patients with primary breast cancer for detection of occult metastatic disease
Source: EMBO Mol Med. 2015 May 18;7(8):1034–47. doi: 10.15252/emmm.201404913 (PMC4551342; doi:10.15252/emmm.201404913)
Supplement: Supplementary file 13 [file emmm0007-1034-sd13.pdf]

EMBO Molecular Medicine

Serial Monitoring of Circulating Tumor DNA in Patients with Primary Breast Cancer for Detection of Occult Metastatic Disease

Eleonor Olsson, Christof Winter, Anthony George, Yilun Chen, Jillian Howlin, Man-Hung Eric Tang, Malin Dahlgren, Ralph Schulz, Dorthe Grabau, Danielle van Westen, Mårten Fernö, Christian Ingvar, Carsten Rose, Pär-Ola Bendahl, Lisa Rydén, ÅBorg, Sofia K. Gruvberger-Saal, Helena Jernström, and Lao H. Saal

Corresponding author: Lao Saal, Lund University

Review timeline:

Submission date:	01 December 2014
Editorial Decision:	26 January 2015
Revision received:	11 March 2015
Editorial Decision:	10 April 2015
Revision received:	14 April 2015
Accepted:	16 April 2015

Transaction Report:

(Note: With the exception of the correction of typographical or spelling errors that could be a source of ambiguity, letters and reports are not edited. The original formatting of letters and referee reports may not be reflected in this compilation.)

Editor: Roberto Buccione

1st Editorial Decision 26 January 2015

Thank you for the submission of your manuscript to EMBO Molecular Medicine. We have now received comments from the Reviewers whom we asked to evaluate your manuscript.

We are very sorry that it has taken much more time than we would have liked to return a decision, but unfortunately we had initial difficulties securing appropriate (and willing) Reviewers and furthermore, in part due to the holiday season overlap, they delivered their evaluations with delay. I trust that the inevitable frustration due to this will be somewhat tempered by the fact that the Reviewers are all supportive of your work, albeit with different nuances and, in my opinion, offer valuable suggestions to improve your manuscript.

Reviewer 1 has important and well-placed concerns that focus on shortcomings in data analysis and presentation. We agree with this assessment and in this respect, please note that EMBO Molecular Medicine now requires a complete author checklist to be submitted with all revised manuscripts (please see further below).

Reviewer 2 is quite positive and offers many valuable suggestions to improve presentation and discussion, which require your action. S/he also addresses technical issues including that the Qiagen kit used might not be entirely appropriate. The Reviewer is also doubtful of your cost estimate and

feels that this issue, important for actual translation to the clinic, requires further in-depth analysis.

Reviewer 3 is also generally positive, especially concerning the technical side of your work. S/he is much more reserved, however, on its clinical relevance. One of the issues raised is that the clinical benefit of earlier metastatic disease detection during follow-up is questionable, also due to the lack of evidence of improved curability. The Reviewer finds especially interesting the potential for early detection (micrometastases) but notes the limited sample size. Finally, Reviewer 3 also mentions some important missing references. Please note that the issue of clinical relevance is very important for us and thus encourage you to carefully address these issues.

In conclusion, while publication of the paper cannot be considered at this stage, we would be pleased to consider a suitably revised submission, provided that the Reviewers' concerns are addressed as outlined above, including with additional experimental data where appropriate.

Please note that it is EMBO Molecular Medicine policy to allow a single round of revision only and that, therefore, acceptance or rejection of the manuscript will depend on the completeness of your responses included in the next, final version of the manuscript.

As you know, EMBO Molecular Medicine has a "scooping protection" policy, whereby similar findings that are published by others during review or revision are not a criterion for rejection. Although I clearly do not foresee such a delay in this case, I do ask you to get in touch with us after three months if you have not completed your revision, to update us on the status. Please also contact us as soon as possible if similar work is published elsewhere.

As mentioned above, EMBO Molecular Medicine now requires a complete author checklist (http://embomolmed.embopress.org/authorguide#editorial3) to be submitted with all revised manuscripts. Provision of the author checklist is mandatory at revision stage; The checklist is designed to enhance and standardize reporting of key information in research papers and to support reanalysis and repetition of experiments by the community. The list covers key information for figure panels and captions and focuses on statistics, the reporting of reagents, animal models and human subject-derived data, as well as guidance to optimise data accessibility. Please make sure that the relevant information is also included in the main manuscript text.

I look forward to receiving you revised manuscript as soon as possible.

***** Reviewer's comments *****

Referee #1 (Remarks):

The authors present data showing that detection of patient-specific rearrangements in plasma can predate clinical diagnosis of recurrent breast cancer.

1. The MS does not precisely state how cases were ascertained i.e. what the denominator was from which cases were selected or whether cases were initially selected and then rejected. The MS should follow strictly the REMARK guidelines and include this data.

2. Were investigators blinded to outcomes and were samples randomized to prevent bias in interpretation? How were progression events detected and scored?

3. The approach is critically dependent on detecting adequate numbers of structural variations in the original breast cancer. How do the 20 samples analysed compare to the broader breast cancer population in terms of the copy-number profiles of their genomes? Do these 20 patients have high rates of genomic instability and are therefore more suited for this type of analysis?

4. The "Selection and Validation of Rearrangements" section does not provide enough details of how rearrangements were selected e.g. from ~92 rearrangements per patient to ~4-6 rearrangements for the assays. These are required for reproducibility and to show how easy this approach would be to implement at scale.

5. It is unclear how the confidence interval for the AUC was determined and what method was used. More detail needs to be given here.

6. The setup for reporting the overall performance of ctDNA in detecting recurrence is biased by the unbalanced structure of the clinical dataset. To call a sample as "positive", the authors accept a positive result from the highest SV for any one of the time points. This biases their test towards a positive result. Given that their cohort contains 14 positive patients and 6 negative then the test will potentially appear to perform better than expected. If prevalence is taken into account when reporting performance, this may mitigate these effects and give a better idea on how this will perform in the clinic. More detailed results need to be reported on how many time points showed a negative result when there was a recurrence present.

Referee #2 (Comments on Novelty/Model System):

This is state-of-the-art ctDNA detection applied to clinically well-annotated and informative sample sets.

Referee #2 (Remarks):

This is a really important proof of concept study on the utility of ctDNA in the follow-up of breast cancer.

Only some specific suggestions:

- In the title, the term "occult recurrent disease" is a little unclear. I would rather say occult metastatic disease, as a disease recurrence is a clinical term. I would also not emphasize the survival aspects ast this point too much in the title nor in the abstract as the sample numbers are too low. The most valuable and convincing data are provided by the individual longitudinal monitoring. The other key point is that high ctDNA counts could indicate early relapse even at diagnosis. Thus, these facts should be emphasized, even though all findings need to be confirmed in larger studies.

- In the technical aspects, there are many critically important contributions, like taking multiple plasma samples from each patient, the sensitivity of the assay (like the amount of plasma) as well as the design of assays for targets that may be selected for, as well as targets that are present at distinct clonal frequencies to allow catching of multiple clones in each tumor. These could be summarized as the major technical features of importance. Perhaps mentioned in the abstract as well.

-Results/Selection and validation of rearrangements: The authors mention that they selected 237 candidate rearrangements to cover a range of copy number states for each tumor. Was the same principle used to select the 4-6 rearrangements for ddPCR assays for each patient? The fraction of rearrangements covered by ddPCR assays here from all rearrangements detected in the tumors (4-6/21-305) was quite small.

-In supplementary Figure S2 patient EM12 shows increase of three ctDNA markers before clinical recurrence at time point 16 months after surgery, but the levels of all three drop to zero (time point 24 months after surgery) possibly due to treatment. Nevertheless, the patient dies at the time point of 34 months after surgery. Thus, based on these markers, the treatment was effective, but in reality there were probably other tumor cell clones with different rearrangements/mutations that caused the death and it would require more markers to reveal such clones.

-How feasible is it to repeatedly design new personalized markers and to probe them with ddPCR? Would this require a new biopsy to find out the new emerging clones? How many ddPCR targets can be monitored at the same time without this becoming impractical?

-Supplementary Figure S2 lacks indication, what are the dashed lines - total ctDNA?

-Figures 3B and 5A should show dashed and dotted lines, respectively, but these were not visible in print.

-The discussion is very well written and recognizes the strengths and weaknesses of the study in a balanced way. It lists many ways that might improve the sensitivity of the developed ddPCR assay that would help to extend the use of ctDNA for prognostics of primary breast cancer patients before surgery. One additional question here concerns the DNA isolation kit:

ctDNA is isolated with QIAamp UltraSens Virus Kit (Qiagen), which is not optimized by Qiagen for extraction of any one type nor all ctDNA types including free DNA, exosomes and viruses even if the name implies it to be virus specific. It has been used to isolate viral nucleic acids (enveloped viruses), so it may extract also DNA from extracellular vesicles such as exosomes in addition to free DNA. Depending on the source of ctDNA (vesicular/free) in the patient plasma, the ddPCR assay sensitivity might be improved by the use of a different kit optimized to isolate that particular type of ctDNA. According to the current protocols for extracellular vesicle isolation, the ctDNA isolation protocol used here will leave small to medium sized vesicles (in addition to free DNA) as possible sources of the ctDNA. Did the authors try other kits and how critical are these for the performance of the assay as a whole.

-Generally, the method monitoring ctDNA seems very good and has outstanding specificity and good sensitivity to detect occult metastasis after surgery. The authors estimate in discussion that it would take 1 month to identify candidate rearrangements and validate personalized ddPCR tests. The reagent costs would be covered by 1000$, which sounds very low. What is the basis for this estimate? If WGS is done, one would presumably likle to do this at sufficient depth to scoremutayions as well, and include germline DNA. Also, if one takes into account the time/work for sequencing data analysis and ddPCR design and optimization, the actual costs are presumably closer to 3000-5000$ for an actual cost of the entire process. Thus, how affordable is the translation to the clinic?

Referee #3 (Remarks):

This manuscript is an original attempt to detect infraclinical metastatic disease in breast cancer. The technique developed here is combining a characterization of primary tumor to detect specific chromosomal rearrangement by low coverage sequencing and a detection of this specific rearrangement by digital PCR in plasmatic circulating tumor DNA.
The authors have demonstrated a very strong correlation between the occurrence of metastatic disease during follow-up and ctDNA detection.
The technical part of this manuscript is of very good quality. The demonstration of the feasibility for this approach would justify the publication of this work. However, from the clinical part of this work several flaws appear:
The number of cases is very limited, as acknowledged by the authors in the discussion (14 with metastatic relapse and 6 without relapse).
The authors should provide a consort diagram explaining the criteria of selection of these cases from their collection. How many breast cancers had plasma collection and follow-up in their institution and could have been available for this study? On which criteria were the 20 cases selected, excluding the information about outcome and plasma availability? To note, there is only one triple negative tumor in this series, in the good prognosis group.
The main purpose of this paper is to demonstrate the possible earlier detection of metastatic disease during follow-up. Currently, there is no evidence of any clinical benefit of this earlier detection for breast cancer in terms or survival or quality of life according to the recommendations for follow-up (Khatcheressian JL, Hurley P, Bantug E, Esserman LJ, Grunfeld E, Halberg F, et al. Breast cancer follow-up and management after primary treatment: American Society of Clinical Oncology clinical practice guideline update. J Clin Oncol. 2013;31(7):961-5.)
Page 3, in the introduction, the assumption that the identification of recurrent disease at the earliest moment could be cured is not substantiated by any data in the literature on breast cancer except in the adjuvant setting. This why there is a consensus about the lack of clinical interest for serum markers like CA15.3 or CEA monitoring during follow-up in breast cancer.
In this study, the authors are not providing any results for comparison with serum tumoral marker which could have been of interest like in Dawson et al (NEJM 2013).
An interesting result is that ctDNA was detectable at time of surgery in four out of 14 patients who

developed distant metastatic relapse. This means it could be possible to detect micrometastatic disease in the adjuvant setting with this technique. The limited number of case in the series does not allow to speculate about the real prognostic value for this detection.
I would recommend to modify the discussion of the paper in order to separate detection of ctDNA at time of diagnosis which can give the opportunity to adapt the adjuvant treatment in the aim of increasing curability rate, and on the other hand to discuss the questionable interest of an earlier detection of metastatic disease. In these cases the narrower time interval between sample and the cost of the analysis would be more difficult to justify.

Minor comment:
The authors could have quoted Rack B et al, JNCI 2014 as the largest study demonstrating prognostic value of CTC in the adjuvant setting.
About mutations in breast cancer, the authors could also refer to Stephens 2012 nature and not only 2009.

1st Revision - authors' response 11 March 2015

Referee #1 (Remarks):

The authors present data showing that detection of patient-specific rearrangements in plasma can predate clinical diagnosis of recurrent breast cancer.

1. The MS does not precisely state how cases were ascertained i.e. what the denominator was from which cases were selected or whether cases were initially selected and then rejected. The MS should follow strictly the REMARK guidelines and include this data.

Reply: First, we thank the reviewer for their thoughtful comments regarding our manuscript and the opportunity to clarify the presentation of our methods and results. We acknowledge the previous version had limitations. We have added a fuller explanation of how the cases were ascertained to the *Methods* section, we have also added a patient flow diagram (new Fig 1A), which is recommended by both REMARK and CONSORT guidelines (see also Reviewer #3's comment). We have revised the manuscript to be in accordance with the REMARK guidelines, and completed the REMARK checklist as well as the EMBO author checklist.

2. Were investigators blinded to outcomes and were samples randomized to prevent bias in interpretation? How were progression events detected and scored?

Reply: These are important points and we have amended the manuscript to clarify how the analyses were performed and the clinical progression events evaluated in the relevant parts of the *Methods*. Regarding the first part of the reviewer's question, we have added the following text to the end of the *Patients* section (page 13): "For all patients included herein, all collected blood sample time-points were analyzed, and study results were blinded to the clinic. In all parts (sequencing, circulating DNA isolation, and ddPCR), patients were analyzed in random order without regard to clinical parameters and the ddPCR data was analyzed in an automatic fashion blinded to outcome and operator (detailed below)."

Regarding clinical progression events, we have also improved the description of the routine systematic clinical follow-up of the patients and how clinical progression was ascertained (page 13): "The 20 patients were diagnosed between November 2002 and May 2007, received the standard of care and were followed according to Swedish National Guidelines as well as additional structured follow-up as part of the BC Blood Study: patients met with a research nurse for study questionnaires (aimed at assessing symptoms and change in medication) and serial blood collection at specified

time points: prior to primary surgery and at approximately 3 to 8-, 12-, 24-, and 36-months follow-up time after primary surgery, and for biennial questionnaires thereafter. This was in addition to the routine clinical follow-up, which for patients not receiving chemotherapy consisted of clinical visits and mammography at follow-up years 1, 2, and 3 after primary surgery, and then by mammographic surveillance in the national screening program; and for patients receiving chemotherapy consisted of a clinical evaluation after completing chemotherapy and followed by yearly clinical visits up through year 5, and then by mammographic surveillance. If any of the follow-up modalities indicated symptoms or signs of metastatic disease, appropriate imaging and confirmatory work-up was performed per standard clinical practice. All cancer therapies are indicated for each patient in Fig E2."

As for detecting molecular progression in an unbiased, uniform, and outcome- and operator-blinded fashion, these methods are described in detail in the sections *Plasma DNA isolation and ddPCR*, *ddPCR data normalization*, *Receiver operating characteristic (ROC) curve analysis*, *Statistical analyses*, and *Logistic regression* (pages 14-16), as well as in the supplementary methods text (now called Expanded View Appendix per EMBO MM guidelines). We have improved the relevant text for greater clarity.

3. The approach is critically dependent on detecting adequate numbers of structural variations in the original breast cancer. How do the 20 samples analysed compare to the broader breast cancer population in terms of the copy-number profiles of their genomes? Do these 20 patients have high rates of genomic instability and are therefore more suited for this type of analysis?

Reply: It is true that our method is only applicable in cases where chromosomal rearrangements are present in the primary tumor and detected by WGS. However, in our experience applying similar low-coverage sequencing of a separate ~40 breast tumor biopsies (part of projects addressing completely different questions), all invasive breast tumors analyzed to date harbor chromosomal rearrangements in similar distributions as the present study, suggesting that our approach is likely to be applicable to nearly all patients with invasive breast cancer. Generally we have found that, while metastatic tumors gain additional rearrangements in comparison to their matched primary tumor, they maintain the majority of rearrangements present in its primary – this was noted in the *Discussion*. Moreover, the numbers of rearrangements detected in the tumors analyzed in the present study are in harmony with the broader breast cancer population as described in other publications (Stephens et al, *Nature* 2009; Banerji et al, *Nature* 2012; and Nik-Zainal et al, *Cell* 2012). The good concordance between our study and these publications is notable, given that the exact number of rearrangements identified in any WGS experiment can be influenced by a many factors including tumor cellularity, fragment size and read length, sequencing chemistry, sequencing depth, alignment algorithm and reference genome, rearrangement identification algorithm, and filtering rules, among others. We have added text to address the reviewer's point in the relevant section at the end of the first *Results* paragraph with these 3 references above.

4. The "Selection and Validation of Rearrangements" section does not provide enough details of how rearrangements were selected e.g. from ~92 rearrangements per patient to ~4-6 rearrangements for the assays. These are required for reproducibility and to show how easy this approach would be to implement at scale.

Reply: We agree and have amended the relevant portions of the *Results* (page 5) and *Methods* sections (Expanded View Appendix) to provide more details regarding the selection and validation strategy. To expand, the strategy was to select ~10 rearrangements per patient tumor for PCR validation in primary tumor DNA and matched normal DNA, and from those which validated, to utilize 4-6 rearrangement assays per patient for ddPCR analysis of ctDNA. The rationale was that we wanted a suitable number of rearrangements to be tested to calculate statistics demonstrating the performance of our pipeline to detect true-positive somatic rearrangements, while at the same time cognizant that we had limited plasma volumes available for these retrospective patients and wanted to run ddPCR replicates and input 4% of the isolated circulating DNA per reaction (limiting us to

the 4-6 assays). Additionally, to account for possible intratumoral heterogeneity and because it is not possible to know *a priori* which rearrangements in the primary tumor will be part of derivative metastatic clone(s), we selected candidate rearrangements such that a range of apparent copy number states (in other words, a range of number of supporting reads) were represented for each patient tumor. We were pleased that this strategy was satisfactory and the approach was manageable: 83% of primer designs were informative, 85% of rearrangements were confirmed somatic, and 93% of probe assays worked in ddPCR (complete breakdown of the PCR validation and ddPCR validation is provided in supplementary Table E4). In a couple instances, notably for patient DF4 the PCR assay failure rate was high and for EM5 a higher number of rearrangements proved to be germline; thus additional assays were designed and tested. Among validated assays, the 4-6 probe assays used per primary tumor were, as above, also selected to reflect a variety of copy number states where possible. In the *Discussion*, we note that the optimal number of rearrangements to analyze per patients and how to select them requires further study (page 10). For example, by comparing many matched primary-metastasis pairs, it may be possible in the future to identify features of primary rearrangements that can predict the likelihood of their maintenance in the metastases.

To further enable implementation at scale, a future task is to computationally automate the design of assays based on SplitSeq analysis of WGS data. This should improve reproducibility and reliability of designs even higher than they already are, and more importantly, should significantly improve the speed and manageability of personalized design at scale. We also believe that our optimized touchdown-PCR strategy contributed to the success rate and specificity of our ddPCR approach, as well as enables scaled implementation. To note, in other projects we also see the great potential and utility of analyzing point mutations in ctDNA (e.g. hotspot mutation assays such as for the 3 *PIK3CA* mutations present in ~25% of primary breast cancers), which may be easier to implement at scale given that they are not personalized. However one drawback for hotspot assays is that they can only be informative for patients with tumors harboring a hotspot mutation (a minority of all breast cancer patients).

5. It is unclear how the confidence interval for the AUC was determined and what method was used. More detail needs to be given here.

Reply: The AUC 95% CI is based on the Clopper-Pearson exact binomial distribution method calculated using the *binom* R package with the function call *binom.confint(x=19, n=20, conf.level=0.95, methods="exact")*. This represents 19 successes in 20 independent binomial trials (since one out of 20 patients, EM3, was misclassified), and resulted in the stated 95% CI of 0.75 to 1.00. We have now added these details to the *Statistical analyses* section of the *Methods*, and we have added details including the function call to the *Statistical analyses* section in the supplementary Expanded View Appendix.

Motivated by the reviewer's question, we assessed the CI with a second method that does not make an assumption about the underlying distribution. To this end, we used the R package *pROC* v1.7.3 (Robin et al, *BMC Bioinformatics* 2011) which uses stratified re-sampling with replacement to compute CIs (ensuring the same number of cases/controls in each replicate as in the original sample). Based on 1 million stratified bootstrap replicates, this resulted in a more optimistic 95% confidence interval of 0.89 to 1.00 for the AUC. Nevertheless, we are reporting the more conservative of these two methods, the original binomial-based CI of 0.75 to 1.00.

6. The setup for reporting the overall performance of ctDNA in detecting recurrence is biased by the unbalanced structure of the clinical dataset. To call a sample as "positive", the authors accept a positive result from the highest SV for any one of the time points. This biases their test towards a positive result. Given that their cohort contains 14 positive patients and 6 negative then the test will potentially appear to perform better than expected. If prevalence is taken into account when reporting performance, this may mitigate these effects and give a better idea on how this will

perform in the clinic. More detailed results need to be reported on how many time points showed a negative result when there was a recurrence present.

Reply: We respectfully disagree that "bias" is the appropriate term to describe the effect of the 2:1 ratio (EM:DF) and the definition of positive sample on our reporting of the overall performance. The overrepresentation of EM patients to DF patients gives us additional power to ascertain the sensitivity of our ctDNA analysis compared to its specificity. Similarly, our definition of a positive patient was intended for maximum sensitivity for early metastasis detection. Given our experience, our limited input material, and considering the specificity of the rearrangement ddPCR approach, the chance of missing DNA fragments (the false negative rate, impacting sensitivity) is a greater problem than false detection (the false positive rate, impacting specificity). Sensitivity and specificity are performance measures that are independent of the composition of the samples and prevalence of events (however with narrower CIs possible with larger group sizes). This is in contrast to other performance measures, such as positive predictive value and negative predictive value, which are not appropriate to report for our study design.

That being said, we do appreciate the reviewer's point. The performance estimates of our method are based on 20 patients with 93 plasma samples of 0.5ml volume analyzed by 4-6 rearrangement assays and thus firmer performance estimates of ctDNA in early breast cancer, and accounting for prevalence, will require larger validation studies with different designs. Our study is an important step forward, but we also acknowledge and emphasize the need for future larger validation studies in the abstract and in the manuscript.

Lastly, to address the last sentence of the reviewer's comment, we now provide additional information on the number of time points with a negative ctDNA result when in fact the patient eventually had metastatic disease (updated Fig 5C).

Referee #2 (Comments on Novelty/Model System):

This is state-of-the-art ctDNA detection applied to clinically well-annotated and informative sample sets.

Referee #2 (Remarks):

This is a really important proof of concept study on the utility of ctDNA in the follow-up of breast cancer.

Reply: We thank the reviewer for their constructive comments and appreciate your recognition of the importance of our study and the value of our rare patient cohort with serially-collected plasma samples.

Only some specific suggestions:

- In the title, the term "occult recurrent disease" is a little unclear. I would rather say occult metastatic disease, as a disease recurrence is a clinical term. I would also not emphasize the survival aspects at this point too much in the title nor in the abstract as the sample numbers are too low. The most valuable and convincing data are provided by the individual longitudinal monitoring.

The other key point is that high ctDNA counts could indicate early relapse even at diagnosis. Thus, these facts should be emphasized, even though all findings need to be confirmed in larger studies.

Reply: We agree. We have made the suggested revisions, replacing “occult recurrent disease” with “occult metastatic disease” in the title and where appropriate throughout the paper, and we have decreased the emphasis on the survival aspects (removed from title, removal of logistic regression p-values from the abstract) and placed more weight in the text on the other aspects of our findings.

- In the technical aspects, there are many critically important contributions, like taking multiple plasma samples from each patient, the sensitivity of the assay (like the amount of plasma) as well as the design of assays for targets that may be selected for, as well as targets that are present at distinct clonal frequencies to allow catching of multiple clones in each tumor. These could be summarized as the major technical features of importance. Perhaps mentioned in the abstract as well.

Reply: Thank you for the kind words. We have attempted to highlight these technical aspects further in the manuscript.

-Results/Selection and validation of rearrangements: The authors mention that they selected 237 candidate rearrangements to cover a range of copy number states for each tumor. Was the same principle used to select the 4-6 rearrangements for ddPCR assays for each patient? The fraction of rearrangements covered by ddPCR assays here from all rearrangements detected in the tumors (4-6/21-305) was quite small.

Reply: Yes, the same principle was followed to select the 4-6 rearrangements for ddPCR analysis of circulating DNA and we have clarified this in the relevant sections of the manuscript. This point is related to the comment #4 from Reviewer #1 and our Reply – for further details, please see our expanded response there and our amendments to the manuscript.

-In supplementary Figure S2 patient EM12 shows increase of three ctDNA markers before clinical recurrence at time point 16 months after surgery, but the levels of all three drop to zero (time point 24 months after surgery) possibly due to treatment. Nevertheless, the patient dies at the time point of 34 months after surgery. Thus, based on these markers, the treatment was effective, but in reality there were probably other tumor cell clones with different rearrangements/mutations that caused the death and it would require more markers to reveal such clones.

Reply: We agree, the Reviewer’s interpretation is certainly plausible. The clone(s) containing these rearrangements may have responded to the therapies given and another clone may have been selected for, which ultimately lead to this patient’s death. Thus, in some instances, monitoring more than 4-6 primary rearrangements to cover all subclones may be needed to further increase sensitivity. Alternatively, better strategies/rules for selecting the rearrangements to monitor may increase the likelihood of monitoring the most “stable” rearrangements that exist in all subclones. As we noted above and in the *Discussion*, primary-metastasis pairs share a surprisingly high percentage of their rearrangements (manuscript in preparation). Another alternative is that larger plasma volumes and use of multiplexing (both mentioned in the *Discussion*) would have enabled detection of some of the selected rearrangements at the 24 month time-point (which was taken under therapy and 10 months before death, so the tumor burden may have been considerably lower). Other possible explanations (albeit unlikely) for EM12’s 24 month time-point result could be blood sampling error at time of collection, mislabeling, improper handling or storage, or technical/lab error.

-How feasible is it to repeatedly design new personalized markers and to probe them with ddPCR? Would this require a new biopsy to find out the new emerging clones? How many ddPCR targets can be monitored at the same time without this becoming impractical?

Reply: As we indicate above, and based on the high degree of shared rearrangements between primary-metastasis pairs (manuscript in preparation), we surmise that "root-level" rearrangements exist which originate early in breast tumorigenesis (see e.g. Chin et al, *Nature Genetics* 2004) and thus are prevalent in most cells of the primary tumor and therefore are likely to be present in clones seeding metastases. By monitoring a number of these root rearrangements, the majority of clones and even emerging clones may still maintain at least one or more of the root rearrangements and thus be traceable. If this is true, then new biopsies would not be required in most cases. This question requires additional studies that are outside of the scope of the present manuscript.

We judge that with increases in efficiency in the assay design step, accomplished by computational automatization of the process, and with robotics, multiplexing, and operating in parallelized plate formats, the approach can be quite scalable. Once a panel of assays is established for an individual, these assays can be re-used for literally hundreds of time-points and reactions over many years at a rather low cost. It is difficult to speculate on exactly how many targets can be monitored in a practical sense, but we are also developing new approaches for multiplexing ddPCR. We make no claims that our approach is necessarily the best approach in all situations or the most scalable. However, given current technological limitations and our experimental limitations such as limited plasma volumes available, our approach was developed and optimized and our results show that ctDNA analysis in early breast cancer is sensitive and specific to detect occult metastasis, is a robust biomarker with great clinical promise, and thus should be evaluated further in this important patient group.

-Supplementary Figure S2 lacks indication, what are the dashed lines - total ctDNA?

Reply: Thank you for noticing this omission. The dashed line is the quantification of total circulating DNA as determined by the control 2p14 control assay (scale on right-side axis). We have added a description of this to the Supplementary Figure S2 legend (renamed to Figure E2).

-Figures 3B and 5A should show dashed and dotted lines, respectively, but these were not visible in print.

Reply: The poor reproduction may be because we embedded the figures inside the manuscript document for review purposes. We have modified all figures to make them more legible and the dashed lines more visible, and now upload all figures as high-resolution source files.

-The discussion is very well written and recognizes the strengths and weaknesses of the study in a balanced way. It lists many ways that might improve the sensitivity of the developed ddPCR assay that would help to extend the use of ctDNA for prognostics of primary breast cancer patients before surgery.

Reply: Thank you for this thoughtful comment. We endeavored to compose a balanced *Discussion*.

One additional question here concerns the DNA isolation kit:

ctDNA is isolated with QIAamp UltraSens Virus Kit (Qiagen), which is not optimized by Qiagen for extraction of any one type nor all ctDNA types including free DNA, exosomes and viruses even if the name implies it to be virus specific. It has been used to isolate viral nucleic acids (enveloped

viruses), so it may extract also DNA from extracellular vesicles such as exosomes in addition to free DNA. Depending on the source of ctDNA (vesicular/free) in the patient plasma, the ddPCR assay sensitivity might be improved by the use of a different kit optimized to isolate that particular type of ctDNA. According to the current protocols for extracellular vesicle isolation, the ctDNA isolation protocol used here will leave small to medium sized vesicles (in addition to free DNA) as possible sources of the ctDNA. Did the authors try other kits and how critical are these for the performance of the assay as a whole.

Reply: We evaluated three methods for isolation of circulating DNA from plasma: the Qiagen UltraSens Virus Kit (cat # 53704), the Qiagen Circulating Nucleic Acid Kit (55114), and the Zymo Research Viral DNA Kit (D3015). In some tests, two purified exogenous short PCR products were used as spike-in controls at known concentrations and measured by ddPCR to estimate the efficiency of isolation. In our hands the UltraSens Virus kit tended to perform the best and gave the highest yields. Therefore, we chose to use this method despite it being the most laborious because we had such limited plasma volumes available to us and wanted to maximize the amount of circulating DNA input in each ddPCR reaction. In future studies where plasma volume may not be as limiting, we do not believe that the method for ctDNA isolation will be critical. We are not aware of comparisons between the various kits as to their efficiency to recover ctDNA with respect to the different sources within plasma (free, exosomal, etc), however we imagine it would be desirable to analyze ctDNA from all cell-free sources for the purpose of a diagnostic assay in order to maximize the assay sensitivity. In this respect, the UltraSens Virus kit may be a better choice than methods that only isolate naked circulating DNA.

-Generally, the method monitoring ctDNA seems very good and has outstanding specificity and good sensitivity to detect occult metastasis after surgery. The authors estimate in discussion that it would take 1 month to identify candidate rearrangements and validate personalized ddPCR tests. The reagent costs would be covered by 1000$, which sounds very low. What is the basis for this estimate? If WGS is done, one would presumably likle to do this at sufficient depth to scoremutayions as well, and include germline DNA. Also, if one takes into account the time/work for sequencing data analysis and ddPCR design and optimization, the actual costs are presumably closer to 3000-5000$ for an actual cost of the entire process. Thus, how affordable is the translation to the clinic?

Reply: The cost estimate is for reagents only and is based on our current in-house costs:

Illumina DNA library prep 1 sample =	$30	€28
HiSeq2500 $29/Gb * 3.2Gb/1x * 5x =	$464	€435
Primers and probe $60 * 5 assays =	$300	€282
ddPCR $4 * 5 assays * 2 reactions =	$40	€38
Blood tube =	$1	€1
Circulating DNA isolation =	$9	€8
Normal DNA isolation =	$5	€5
Other (PCR, plastics, consumables) [generous] =	$100	€94
Total =	$949	€891

This is sufficient for detection of chromosomal rearrangements in the primary tumor as performed in our study but not for calling mutations, and omits sequencing the normal DNA as most common normal structural variants can now be filtered based on in-house and public data. This is also an estimate for one plasma time-point. Each additional time-point analyzed will cost approximately €50 in reagents. These estimates do not account for labor and other intangible costs, but exactly how much additional costs there are will depend on many factors such as scale of operation, administration and overhead, local labor cost, labor skill and efficiency, equipment depreciation, and so forth. Due to the difficulties in estimating these intangibles, we do not include these additional costs in our estimate. Nevertheless, the total cost appears to be within reason for a clinical test and

affordable should such analyses be one day proven to lead to reduced overall healthcare cost (e.g. savings from reducing overtreatment, fewer interventions based on false-positives such as imaging) or lead to improved outcomes and quality of life. In the revision we now report the estimated cost in Euros, the local currency of EMBO.

Referee #3 (Remarks):

This manuscript is an original attempt to detect infraclinical metastatic disease in breast cancer. The technique developed here is combining a characterization of primary tumor to detect specific chromosomal rearrangement by low coverage sequencing and a detection of this specific rearrangement by digital PCR in plasmatic circulating tumor DNA.

The authors have demonstrated a very strong correlation between the occurrence of metastatic disease during follow-up and ctDNA detection.

The technical part of this manuscript is of very good quality. The demonstration of the feasibility for this approach would justify the publication of this work.

Reply: We thank the reviewer for a very careful reading of our manuscript and for bringing up important points that have helped us to improve the paper.

However, from the clinical part of this work several flaws appear:

The number of cases is very limited, as acknowledged by the authors in the discussion (14 with metastatic relapse and 6 without relapse).

The authors should provide a consort diagram explaining the criteria of selection of these cases from their collection. How many breast cancers had plasma collection and follow-up in their institution and could have been available for this study? On which criteria were the 20 cases selected, excluding the information about outcome and plasma availability? To note, there is only one triple negative tumor in this series, in the good prognosis group.

Reply: Our sample is limited in size, however the combination of such tumor and blood material with long-term clinical follow-up is rather unique and valuable. Despite the limited sample, our results show great promise for serial ctDNA analysis in early breast cancer, as has been shown in metastatic breast cancer (Dawson et al, *NEJM* 2013). We have revised the *Methods* and now more clearly describe the patient selection process and also include a new Fig 1A patient flow diagram as recommended by both CONSORT and REMARK (see also Reviewer #1's comment): "Patients enrolled in the Breast Cancer and Blood Study (BC Blood, Sweden) (Borgquist et al, 2013), an ongoing prospective study at Lund University since 2002, were included in the present investigation for retrospective analysis of ctDNA. As shown in Fig 1A, patients were identified based on the following criteria: non-metastatic (stage I-III) breast cancer at initial diagnosis who received no neoadjuvant therapy, availability of frozen primary tumor specimen, frozen pre-surgery and two or more follow-up plasma samples collected during clinical course, and either clinically-detected distant metastasis 1 to 6 years after diagnosis (termed eventual-metastatic [EM] patients) or long-term disease-free survival >7 years at last follow-up (termed DF patients). Out of 725 patients assessed, 24 EM and 63 DF patients passed eligibility requirements. From these, 20 patients were randomly selected 2:1 with respect to EM:DF categories. This sample size with multiple time-points per patient was considered to be sufficient to demonstrate the feasibility of ctDNA monitoring and test the hypothesis that occult metastasis can be detected by ctDNA analysis. Fourteen EM patients (first metastasis detected clinically at 14 to 61 months following diagnosis, median 20 months) and

6 DF patients (disease-free at last follow-up, 109 to 113 months after diagnosis, median 110 months) were studied (Table 1 and Fig 1)."

Since CONSORT is aimed towards reporting for clinical trails, we have elected to follow the REMARK checklist.

The main purpose of this paper is to demonstrate the possible earlier detection of metastatic disease during follow-up. Currently, there is no evidence of any clinical benefit of this earlier detection for breast cancer in terms or survival or quality of life according to the recommendations for follow-up (Khatcheressian JL, Hurley P, Bantug E, Esserman LJ, Grunfeld E, Halberg F, et al. Breast cancer follow-up and management after primary treatment: American Society of Clinical Oncology clinical practice guideline update. J Clin Oncol. 2013;31(7):961-5.)

Page 3, in the introduction, the assumption that the identification of recurrent disease at the earliest moment could be cured is not substantiated by any data in the literature on breast cancer except in the adjuvant setting. This why there is a consensus about the lack of clinical interest for serum markers like CA15.3 or CEA monitoring during follow-up in breast cancer.

In this study, the authors are not providing any results for comparison with serum tumoral marker which could have been of interest like in Dawson et al (NEJM 2013).

Reply: We agree, there currently is no evidence for a clinical benefit of earlier detection and we have added the suggested reference to the *Introduction*. However, we would argue that the assumption has not been proven to be false. Our hypothesis, and one shared by others (see for example Lippman and Osborne, *NEJM* 2013) is that prior modalities such as serum markers may have lacked the sensitivity, specificity, and pharmacodynamic properties for the earliest and most accurate molecular discrimination of disease status. Thus, it is possible that no benefit has been seen previously because prior modalities are not detecting occult disease early enough and/or not accurately enough. Our proof-of-concept results are an important stepping stone that argue for further evaluation of ctDNA monitoring in larger validation studies in early breast cancer to test for example for MRD/cure, detect occult metastatic disease, and steer therapy.

An interesting result is that ctDNA was detectable at time of surgery in four out of 14 patients who developed distant metastatic relapse. This means it could be possible to detect micrometastatic disease in the adjuvant setting with this technique. The limited number of case in the series does not allow to speculate about the real prognostic value for this detection.

I would recommend to modify the discussion of the paper in order to separate detection of ctDNA at time of diagnosis which can give the opportunity to adapt the adjuvant treatment in the aim of increasing curability rate, and on the other hand to discuss the questionable interest of an earlier detection of metastatic disease. In these cases the narrower time interval between sample and the cost of the analysis would be more difficult to justify.

Reply: The association between pre-operative ctDNA level and outcome is based on a low number of observations and is not significant, but we agree it is intriguing and should be evaluated in future studies. We have initiated prospective studies in the neoadjuvant/adjuvant setting that may shed light on this question, however considerable follow-up time will be required.

As suggested, we have re-written portions of the *Discussion* to better separate and qualify the various potential uses of ctDNA monitoring.

Minor comment:

The authors could have quoted Rack B et al, JNCI 2014 as the largest study demonstrating prognostic value of CTC in the adjuvant setting.

About mutations in breast cancer, the authors could also refer to Stephens 2012 nature and not only 2009.

Reply: Thank you for pointing out these references; they have been added now to the revised manuscript.

2nd Editorial Decision 10 April 2015

Thank you for the submission of your revised manuscript to EMBO Molecular Medicine. We have now received the enclosed reports from the referees that were asked to re-assess it. As you will see the reviewers are now supportive and I am pleased to inform you that we will be able to accept your manuscript pending the following final amendments:

1) Every published paper now includes a 'Synopsis' to further enhance discoverability. Synopses are displayed on the journal webpage and are freely accessible to all readers. They include a short standfirst as well as 2-5 one sentence bullet points that summarise the paper. Please provide the synopsis including the short list of bullet points that summarise the key NEW findings. The bullet points should be designed to be complementary to the abstract - i.e. not repeat the same text. We encourage inclusion of key acronyms and quantitative information. Please use the passive voice. Please attach this information in a separate file or send them by email, we will incorporate it accordingly. You are also welcome to suggest a striking image or visual abstract to illustrate your article. If you do please provide a jpeg file 550 px-wide x 400-px high.

2) It is very important that you submit your droplet digital PCR data and normalized data to a publicly accessible database prior to acceptance. Please read carefully our Data Deposition guidelines at http://embomolmed.embopress.org/authorguide#datadeposition.

Please submit your revised manuscript within two weeks. I look forward to seeing a revised form of your manuscript as soon as possible.

***** Reviewer's comments *****

Referee #1 (Remarks):

Excellent clarifications and improvements. No further comments.

Referee #2 (Remarks):

Comments addressed appropriately.

Referee #3 (Remarks):

No further comments.

2nd Revision - authors' response 14 April 2015

We are pleased that our explanations and revisions were acceptable, and that *EMBO Molecular Medicine* is willing to accept our manuscript following minor amendments. Please find enclosed our revised manuscript that address the final issues:

• A synopsis with bullet points is attached as well as a suggested image in jpg format. In addition, we provide a draft for the “paper explained” section.

• The ddPCR data are now available from the public Dryad Digital Repository with permanent digital object identifier doi:10.5061/dryad.b6928 (manuscript text updated accordingly).

• The supplementary information is provided as an expanded view appendix PDF (containing the supplementary methods and new table of contents). In addition, as separate files, there are the expanded view figures (E1, E2) and expanded view tables (E1 to E7, in Excel format). Due to the dimensions of these supplementary figures and tables, we could not combine them all into a single PDF together with the methods without compromising their usefulness. Therefore, we hope that the supplementary figures and tables may be provided to readers as individual files in their source formats. Please advise if this is a workable solution for the journal or if you have another suggestion.

We thank you, the editorial team, and the peer reviewers for a first-rate submission and review process
